# Changing socioeconomic inequalities in the incidence and case fatality rates of COVID-19 in Germany, March 2020 through May 2022: an ecological study

**DOI:** 10.1186/s12889-025-24625-9

**Published:** 2025-10-02

**Authors:** Sarah C. Kramer, Laura Andrea Barrero Guevara, Matthias an der Heiden, Benjamin Wachtler, Matthieu Domenech de Cellès

**Affiliations:** 1 Max Planck Institute for Infection Biology, Infectious Disease Epidemiology group, Charitéplatz 1, Campus Charité Mitte, 10117 Berlin, Germany; 2 Institute of Public Health, Charité – Universitätsmedizin Berlin, Charitéplatz 1, 10117 Berlin, Germany; 3 Department of Infectious Disease Epidemiology, Robert Koch Institute, Nordufer 20, 13353 Berlin, Germany; 4 Department of Epidemiology and Health Monitoring, Robert Koch Institute, Nordufer 20, 13353 Berlin, Germany

**Keywords:** COVID-19, SARS-CoV-2, Socioeconomic position, Socioeconomic deprivation, Ecological study, Generalized additive models, Social determinants of health

## Abstract

**Background:**

Socioeconomic disparities in COVID-19 burden were widely observed during the pandemic’s early waves, including in Germany, but studies on whether these inequalities have persisted or changed as the pandemic progressed are lacking.

**Methods:**

We used an ecological study design to assess the relationship between a range of demographic, socioeconomic, and healthcare-related predictors and COVID-19 impact in Germany. Specifically, we fit generalized additive models to cumulative, district-level (*n* = 400) COVID-19 incidence and case fatality rates (CFRs) for each of the first five pandemic waves, which covered the period from March 2020 through May 2022.

**Results:**

We find that associations between socioeconomic deprivation and COVID-19 impact evolved over time. Specifically, districts with higher levels of deprivation experienced lower incidence initially, but higher incidence beginning in the second half of wave 1 and persisting through wave 3. Meanwhile, more deprived districts experienced higher CFRs initially as well as during waves 3 through 5, but lower CFRs during the second half of wave 1. Finally, during the first four waves, we find that district-level CFRs scaled superlinearly with incidence, suggesting that the risk of death increased with incidence. This relationship was particularly strong during the first pandemic wave.

**Conclusions:**

The association between socioeconomic position and COVID-19 impact in Germany has been complex, with patterns changing in intensity and direction over time. Continued monitoring of socioeconomic inequalities in COVID-19 impact, in particular at the individual level, is needed to better understand if and how inequalities continue to persist. Such monitoring will be instrumental in informing more equitable control strategies.

**Supplementary Information:**

The online version contains supplementary material available at 10.1186/s12889-025-24625-9.

## Background

Since the beginning of the coronavirus disease 2019 (COVID-19) pandemic, caused by the severe acute respiratory syndrome coronavirus 2 (SARS-CoV-2), substantial inequalities in the pandemic’s impact by socioeconomic position (SEP) have been reported [1–8]. These inequalities have been identified by both ecological and individual-level studies, as well as on a range of spatial scales, including within cities [6–8], within countries [1, 3–5], and internationally [2]. Most commonly, studies have shown that those with lower SEP have experienced higher COVID-19 incidence and severity throughout much of the pandemic [9–11]. Bambra suggests that these patterns arise along four major pathways; specifically, inequalities in exposure, transmission, susceptibility, and treatment due to SEP drive resulting inequalities in COVID-19 outcomes [12]. Here, unequal exposure and transmission due to factors like a lack of opportunities for remote work and crowded living conditions drive higher rates of infection among those with lower SEP. Meanwhile, material deprivation, higher prevalence of chronic health conditions, and reduced access to high-quality healthcare lead to disparities in susceptibility and treatment, which in turn contribute to unfavorable outcomes among those infected. Notably, similar disparities have been observed in past pandemics involving respiratory pathogens, including the 1918 and 2009 influenza pandemics [13, 14], as well as during more regular outbreaks of a wide range of infectious diseases [15–17]. Understanding where these inequalities exist is critical if we are to ensure an equitable outbreak response, both during future epidemic waves and endemic spread.

Research during the first pandemic waves has suggested that similar inequalities are also at play in Germany. Interestingly, several groups have reported that COVID-19 incidence was substantially higher in regions with higher SEP during the first weeks of the pandemic’s first wave [18, 19]. This is likely due to the fact that SARS-CoV-2 was first introduced to Germany by those returning from international ski trips, who were likely to be relatively well-off [20]. As the pandemic spread and lockdown measures were instituted, these initial conditions gradually became less important, and factors determining one’s ability to comply with social distancing recommendations, such as SEP, began to play much more of a role [19]. As a result, the initial trends began to disappear by the end of the first wave in southern and western regions of Germany, where the first wave dominated [18, 19]. A similar pattern was observed during the second wave for the whole of Germany, and, by midway through the second wave, regions with lower average SEP were consistently experiencing higher incidence and mortality rates than less deprived regions across the country [21, 22].

Although previous studies have assessed the association between SEP and COVID-19 outcomes in Germany for the first two pandemic waves [18, 19, 21–25], there is little understanding of how these associations have evolved during subsequent waves. Furthermore, the bulk of past work focuses on incidence (cases per population) and mortality (deaths per population), but does not directly evaluate the association between SEP and the risk of severe outcomes resulting from SARS-CoV-2 infection. Finally, existing studies have typically not accounted for the spatial dependence between neighboring and highly-connected regions. This is an issue because spatially correlated data are not independent, and analyses ignoring spatial dependence may therefore result in overly-confident effect estimates.

Here, we evaluate how district-level COVID-19 incidence and case fatality rates (CFRs) in Germany varied according to several demographic and socioeconomic predictors during each of the first five waves of the pandemic, using an ecological study design. Specifically, we fit the data using generalized additive models (GAMs) [26], a method capable of identifying nonlinear and non-monotonic associations with much more flexibility than standard regression methods. Our work expands on previous studies in a number of important ways. First, by including the first five pandemic waves, we assess how relationships between district-level socioeconomic deprivation and COVID-19 outcomes have changed over time. Furthermore, by analyzing patterns in CFRs by deprivation, we directly assess whether deprivation is associated with COVID-19 lethality. Finally, by including a two-dimensional smooth of latitude and longitude, our statistical models control for spatial dependence between different regions in Germany. Based on past results and on broader theory, we expect that there will be substantial differences in regional incidence and CFR by various indicators of demography, healthcare, and SEP, and that these relationships will evolve over the course of the pandemic.

## Methods

### Study period

For our analyses, we divided the pandemic into individual waves. We defined the start and end points of each pandemic wave based both on decisions made in the literature [27], and on the progression of the pandemic at the country level (Figure S1; see Supplementary Materials for details). Specifically, we defined each wave as follows:

1. Week 9 (2020) - Week 20 (2020).
2. Week 40 (2020) - Week 8 (2021).
3. Week 9 (2021) - Week 21 (2021).
4. Week 32 (2021) - Week 51 (2021).
5. Week 52 (2021) - Week 21 (2022).

We then calculated the cumulative number of cases and deaths in each district throughout each wave. Based on preliminary results suggesting that the direction of the association between incidence and SEP changed midway through the first wave, we further divided wave 1 into two partial waves, with the first partial wave (wave 1.1) including weeks 9 through 14 and the second (wave 1.2) including weeks 15 through 20 of 2020 (see Supplementary Materials for methodological details). Additionally, details of a sensitivity analysis where all waves were split into two partial waves can be found in the Supplementary Materials (Figure S7).

A timeline of the COVID-19 pandemic in Germany, including information on the government response to the outbreak as well as testing and vaccination policy over time, can be found in the Supplementary Materials (“Timeline of the COVID-19 Pandemic in Germany” and Figure SS2). Briefly, the German government instituted two major lockdowns in response to the pandemic, one during wave 1 and the other during wave 2 (Figure S2A). Various control strategies were enforced throughout the study period, with measures generally being stricter during the earlier waves.

### Study design

Many previous studies on the socioeconomic predictors of COVID-19 impact have focused on incidence (cases per population) and mortality (deaths per population) [9]. However, increasing mortality rates do not necessarily indicate an increased risk of death due to infection. Indeed, if the risk of death is uniform, mortality rates will still be higher in regions with higher incidence. In order to separately assess the association of our predictors with the probability of infection and with the probability of death due to SARS-CoV-2, we instead chose incidence and CFRs as our outcomes of interest.

We employed an ecological study design at the district level in Germany. Germany is made up of a total of 400 districts (“*Landkreise*”), ranging in size from 34,000 to 3.6 million residents (median = 156,000); these districts are divided between 16 federal states (“*Bundesländer*”). Therefore, the dependent variables in our models were the cumulative number of laboratory-confirmed cases or deaths in each pandemic wave, by district. This spatial resolution was chosen as it was the smallest spatial resolution at which data on COVID-19 outcomes are publicly available. A single district, which resulted from the merging of two prior districts during the pandemic, was removed from the analysis, leaving 399 districts.

To assess the relationships between district-level COVID-19 incidence and CFRs and our demographic, healthcare, and socioeconomic variables of interest, we used generalized additive models (GAMs) [26, 28]. By fitting dependent variables to smooth functions of the independent variable data, GAMs are able to flexibly capture nonlinear and non-monotonic relationships between variables that are not so easily modeled using standard generalized linear modeling (GLM) methods. Because GAMs are based on a penalized likelihood method, they are skilled at allocating the appropriate amount of “wiggliness” needed for each smooth, while simultaneously avoiding overfitting. A final advantage is that GAMs allow for the inclusion of two-dimensional smooth functions of latitude and longitude, thereby efficiently controlling for lack of spatial independence in spatial data.

### Data collection and formatting

#### COVID-19 Data

Data on the number of COVID-19 cases and deaths among residents of each district in Germany were obtained from the Corona Data Platform [29], a database maintained through early 2025 which made a wide range of data on COVID-19 in Germany available to the public. Cases and deaths are included in the data if infection with SARS-CoV-2 is confirmed by nucleic acid amplification test (e.g., PCR) or viral isolation, regardless of symptoms [30]. For deaths, this means that both deaths judged to be directly caused by SARS-CoV-2 infection, as well as deaths where the role of infection on mortality is unclear, are reported. Testing by healthcare providers is particularly recommended for patients with severe respiratory symptoms, loss of smell or taste, or recent close contact with a confirmed case. Additionally, PCR tests could be accessed by the general public at testing centers throughout much of the pandemic, although tests were not always free, and it is likely that the majority of infected individuals instead made use of rapid antigen tests, which were cheaper and yielded results much more quickly. More details on SARS-CoV-2 test policy and availability throughout the pandemic in Germany can be found in the Supplementary Materials. Since regional and local public health departments are required by law to report confirmed cases and deaths due to SARS-CoV-2 to the Robert Koch Institute (RKI), Germany's federal institute for public health, we expect that these data are high-quality [18, 22].

The raw data include the daily number of new laboratory-confirmed cases and deaths in each of six age groups (0–4, 5–14, 15–34, 35–59, 60–79, and 80+). We note here that deaths are not reported by the date on which the respective death occurred, but rather by the date on which the corresponding case was first reported to public health authorities. Given that COVID-19 is substantially more likely to lead to severe disease and death in older individuals [31], we controlled for the difference in age distribution between districts using direct age standardization [32], using the age groups listed above, and with the population of Germany acting as the reference population (see Supplementary Materials). Population data from 2020 were also obtained from the Corona Data Platform.

#### Demographic and Socioeconomic Data

We measure district-level socioeconomic position using the German Index of Socioeconomic Deprivation (GISD), which measures the relative level of socioeconomic deprivation across Germany, such that higher values of the index indicate lower SEP (Figure S3) [33]. The index compiles data on various indicators of regional educational attainment, employment, and income. The most recent version of the index uses data from 2019. More information on the development of this index can be found in the Supplementary Materials and in [33].

District-level data on additional demographic, healthcare, and socioeconomic predictors (see “Variable Selection” below) were obtained from the database “Indicators and Maps for Spatial and Urban Development” (INKAR; “*Indikatoren und Karten zur Raum- und Stadtentwicklung*”) [34], a publicly-available repository published by the Federal Institute for Research on Building, Urban Affairs and Spatial Development and containing regional data on a wide variety of themes. Most data were collected in 2020, with the exception of data on beds in long-term care facilities, which were collected in 2019, and on employment sector, which were collected in 2017.

#### Vaccination Data

Because vaccinated individuals are less likely to be infected with SARS-CoV-2, and to experience severe disease in the case of an infection [35], it is important to control for district-level vaccination rates. In Germany, vaccination against SARS-CoV-2 began on 26 December 2020 (during wave 2). Residents were sorted into priority groups, such that the vaccine was first made available to those at highest risk. More details on vaccination policy over time in Germany can be found in the Supplementary Materials.

The RKI collects district-level data on COVID-19 vaccination. However, these data are collected based not on individuals’ home districts, but instead based on the district in which they were vaccinated [36]. This means that some districts’ reported vaccination rates severely underestimate the true vaccination rates of people living there, while other districts’ data substantially overestimate vaccination rates, with reported rates sometimes exceeding 200% [36]. This is particularly noticeable for large urban areas and their surrounding districts, where people may travel into nearby cities to get vaccinated. To estimate district-level vaccine coverage, we use average vaccination rates for 33 labor market regions throughout Germany, and apply these rates to all districts located within a region, as described in Koslow et al. [36–38]. This results in more realistic estimates of vaccination rates, while still maintaining local differences by avoiding averaging over large spatial areas. More specifically, we focus on the proportion fully vaccinated against SARS-CoV-2 (defined in the Supplementary Materials).

### Choice of predictor variables

In addition to the GISD, we searched the INKAR database for possible predictors of interest. Variables were only selected if there was a clear, hypothesized mechanism linking the variable to the probability of infection with SARS-CoV-2 or death due to COVID-19 (Figure S4). This approach was taken to avoid the inclusion of variables potentially correlated with COVID-19 incidence or CFR, but unlikely to be drivers of public health outcomes.

A summary of the variables included in models of COVID-19 incidence and CFRs can be found in Table 1. For models of incidence, although the age standardization of the data should control for any individual-level impact of age on infection, it is possible that an increased proportion of children or working-age adults at the district level could lead to higher rates of infection, given that these populations attend school and work, and may also be involved in more social activities that involve larger groups [39, 40]. Both higher population density and smaller living areas could lead to crowding, which could facilitate SARS-CoV-2 transmission [41, 42]. People working in either the service or production sectors are unlikely to be able to work remotely, potentially leading to greater exposure to SARS-CoV-2 [23, 24, 43]. Furthermore, working conditions in these fields often involve high levels of close contact with coworkers or clients, which could also lead to increased rates of infection [43–45]. Finally, since COVID-19 has been observed to spread rapidly through care homes [46], an increase in care home beds per capita may be associated with higher infection rates.

**Table 1:** Demographic, socioeconomic, and healthcare variables included in fitted models

Variable	Name in Models	Description	Included in Model of:		Source
			Incidence	CFR	
GISD	GISD_score	German Index of Socioeconomic Deprivation	x	x	(32)
% Aged < 18	perc_lessthan18	Percent of the population aged less than 18	x		(33)
% Aged 18–64	perc_18to64	Percent of the population aged 18–64	x		(33)
Population Density	pop_dens	Population density (100’s of people per square kilometer of land used for settlement and transportation)	x	x	(33)
Living Space	living_area	Average living space per person in square meters	x		(33)
% Workers Service	perc_service	Percent of workers employed in person-related service jobs	x		(33)
% Workers Production	perc_production	Percent of workers employed in production-oriented jobs	x		(33)
Hospital Beds	hosp_beds	Number of hospital beds per 1000 population		x	(33)
Care Home Beds	care_home_beds	Number of spots in long-term care facilities per 10,000 population	x	x	(33)

For CFRs, a larger number of hospital beds per capita may be expected to reduce hospital crowding, potentially reducing CFRs [47]. More hospital beds could also indicate a greater number of high-level care providers, who could have provided more specialized care to severe cases. Meanwhile, since the populations living in care homes, including the elderly and people with a range of chronic health conditions, tend to be populations at high risk for severe COVID-19 outcomes [46, 48], we may expect a larger number of care home beds per capita to be linked to higher CFRs. In addition to these variables, population density was included to control for any potential unmeasured differences between urban and rural areas that might lead to different CFRs due to COVID-19.

Average living area per person was ultimately removed from consideration, as it was highly correlated with population density (Kendall’s tau = −0.49), and its inclusion did not consistently improve model fit. All remaining predictors of interest were included in the models of COVID-19 incidence and CFR. No additional variable selection procedures were used.

### Model building

For models of incidence, the outcome variable of interest was the cumulative number of cases per district over the course of each wave, with the logarithm of district population sizes taken as an offset, such that our outcome of interest is the per capita incidence rate. For models of CFR, cumulative deaths over each wave were the outcome variable, with offset equal to the logarithm of the cumulative number of cases over the same wave. Because deaths in the original data were reported by the date on which the corresponding case was originally reported, and not the date on which the death occurred (see “COVID-19 Data” above), there was no need to account for a delay between cases and deaths. Both incidence and CFRs were modeled using negative binomial regression with a log-link, as models fit using a Poisson distribution showed evidence of overdispersion.

In addition to the predictors discussed above, fitted models also included the following terms. First, to account for spatial dependence, we smoothed over the latitude and longitude of the district centroids (or a nearby point when centroids were located outside the district) using a two-dimensional Duchon spline basis [26]. Centroids were calculated based on district-level spatial data published by the Federal Agency for Cartography and Geodesy (© GeoBasis-DE/BKG (2022)) [49]. Results of a sensitivity analysis where this spatial smooth was excluded can be found in the Supplementary Materials. To control for potential differences in the size of the susceptible population, we also included smooths of (1) COVID-19 incidence in the 26 weeks (roughly 6 months) prior to the current wave, and (2) the proportion of the population that was fully vaccinated as of two weeks prior to the wave’s midpoint. Since vaccination in Germany did not begin until more than halfway through the second wave, vaccination rates were only included in models of waves 3 through 5. Finally, to account for unmeasured state-level variability in surveillance, data collection, and public health control measures, we included a state-level random effect.

For models of CFR only, we also included the cumulative incidence over the course of the current wave as a predictor. This was done to test whether the number of deaths scaled linearly with the number of cases, or whether increased incidence might be linked to increased CFR, either because of the increased burden on the healthcare system, or because of a corresponding increase in the population-level force of infection [50].

When fitting GAMs, it is necessary to specify the dimension of the basis underlying the desired smooth (“k”), which places an upper bound on the complexity of the association [26]. Choosing a proper value for k is particularly important for the spatial smooth, as relatively large values may be necessary to model complex, nonlinear spatial patterns, but values that are too large can lead to overfitting and slow computation times. To determine the ideal range of k-values for each model, we fit models of incidence and CFR for each wave using k-values ranging from 10 to 150, and selected tentative values based on the Akaike (AIC) and Bayesian (BIC) information criteria of the resulting models. These values were then fine-tuned to ensure that no spatial autocorrelation remained and no significant issues with model fit occurred (see “Model Assessment” below). For the state-level random effect, k was set to 16, the number of states in Germany.

Interactions between predictors were included in the models if there was a plausible mechanism by which the interaction could influence either incidence or CFR, and if inclusion of the interaction noticeably improved model fit. Specifically, we considered potential interactions between socioeconomic deprivation, age, and population density, as well as between incidence and hospital beds per capita.

The final equations for all models of incidence were:

1 $$\begin{aligned}&\:cases\_wav{e}_{x}\:\sim\:NB({\mu\:}_{x},\:{\theta\:}_{x})\\ & \:log\left({\mu\:}_{x}\right)={{\alpha\:}_{0}}_{x}+\:s(long,\:lat)+\:1\:|\:bl+s(perc\_18to64)\\ &+s(perc\_lessthan18)+s(care\_home\_beds)\\&+s(GISD\_score)+s(pop\_dens)\\ &+s(perc\_service)+s(perc\_production)\\&+s(incidence\_pre\_wav{e}_{x})+s(vacc\_wav{e}_{x})\\ &+offset(log\left(pop\right)) \end{aligned}$$

where “s” refers to a smooth term of the specified variables. The observed case count during wave *x* was modeled using a negative binomial model with mean *µ*_*x*_ and dispersion parameter *θ*_*x*_, where *µ*_*x*_ is defined as above and *θ*_*x*_ is estimated during model fitting. In other words, observed case counts are assumed to come from a distribution with expectation *µ*_*x*_ and variance $\:{\mu\:}_{x}+\frac{{\mu\:}_{x}}{{\theta\:}_{x}}$. Incidence during the 26 weeks prior to a given wave *x* is indicated by “incidence_pre_wave_x_,” while estimated vaccination rates during wave *x* are indicated by “vacc_wave_x_” (waves 3–5 only). The spatial smooth is represented by “s(long, lat)” and the state-level random effect by “1 | bl.” The term $\:{\alpha\:}_{{0}_{x}}$ is the model intercept. All other variables are named as in Table 1 and in the associated model code (see “Software” below).

The final equations for all models of CFR were:

$$\begin{aligned}&\:deaths\_wav{e}_{x}\:\sim\:NB({\nu\:}_{x},\:{\tau\:}_{x})\\ & \:log\left({\nu\:}_{x}\right)={{\beta\:}_{0}}_{x}+\:s\left(long,\:lat\right)+1\:|\:bl+s(hosp\_beds)\\ &+s(care\_home\_beds)+s(GISD\_score)\\&+s(pop\_dens)+s(incidence\_wav{e}_{x})\\ &+s(incidence\_pre\_wav{e}_{x})+s(vacc\_wav{e}_{x})\\ &+offset(log(cases\_wav{e}_{x})) \end{aligned}$$

where observed deaths are assumed to be a draw from a negative binomial distribution with mean *ν*_*x*_ and dispersion *τ*_*x*_. Incidence during a given wave *x* is indicated by “incidence_wave_x_,” $\:{\beta\:}_{{0}_{x}}$ is the intercept, and all other terms are as described above. For the model of CFR in wave 3 only, an interaction effect between GISD score and population density was also included.

In addition to models including all variables discussed above, we also fit “null” models for each outcome and wave, which included the spatial smooth, state-level random effects, current and past incidence, and vaccination rates, but did not include any demographic or socioeconomic predictors. This was done to evaluate the extent to which the inclusion of demographic and socioeconomic variables improved model fit.

Further details on the model-building process can be found in the Supplementary Materials.

### Model assessment

To confirm good-quality fit to the data, we calculated and inspected model residuals (see Software below for details). We also checked for spatial autocorrelation in the residuals using the Moran’s I statistic, which quantifies the degree to which data are more or less spatially clustered than a variable randomly distributed in space [51].

Full-fitted models including demographic and socioeconomic predictors were compared to “null” models by comparing the deviance explained. To assess the association between each predictor of interest and COVID-19 incidence and CFRs, we calculated the effect of each predictor conditional on all other variables, while holding latitude and longitude constant at the values corresponding to the district with observed outcome nearest to the mean outcome for that wave, and all other variables constant at their mean values, and predicting incidence and CFR from the fitted models. To allow for easier comparison of the strength of associations by wave, we rescaled the predictions by dividing by the mean predicted value for each variable and wave. The resulting values indicate the multiplicative extent to which increasing or decreasing the variable of interest changes incidence or CFR, relative to the mean.

### Spatial analysis

In addition to controlling for the lack of spatial independence in the data, smooths of latitude and longitude can be directly analyzed to gain insight into the underlying spatial distribution of the dependent variables. Here, we use permutation-based partial Mantel tests [52] with Spearman rank correlation coefficients to assess whether the underlying spatial patterns in COVID-19 incidence are associated with the magnitude of commuting flows between districts, after controlling for geographic distance. District-level differences in model-predicted incidence were obtained by extracting the marginal effects of latitude and longitude while holding all other variables constant at their mean values, as described above. Importantly, these values represent differences in the underlying spatial distribution of incidence after controlling for all other variables in the model, rather than differences in observed incidence. Data on the work and home districts of commuters in 2020 were obtained from the Federal Employment Agency [53]; where exact numbers of commuters between districts were very low and therefore not reported, estimates generated by Kühn et al., assuming that commuting rates were proportional to population counts in the home district, were used [37, 38]. Significance levels were based on 1000 permutations of the incidence and CFR difference matrices.

### Software

All analyses were conducted in R version 4.4.0 [54]. GAMs were fit using the package mgcv (version 1.9.1) [26] and visualized using ggplot2 (version 3.5.1) [55]. Goodness-of-fit was assessed using the DHARMa package (version 0.4.6) [56], which uses simulation methods to evaluate model residuals. All code used for this project is freely available on GitHub at: https://github.com/sarahckramer/covid_ses.

## Results

### Descriptive results

Cumulative, age-standardized incidence and CFRs for each wave of COVID-19 in Germany are shown in Table 2 and Figure S5. Cumulative incidence by wave tended to increase throughout the pandemic, with low incidence in wave 1 and moderate incidence during the subsequent three waves. By far the highest incidence was observed during wave 5, defined as the first wave during which the Omicron variant dominated, and where average incidence was over six times that observed in the previous wave. In contrast, CFRs were highest in the first two waves, and lowest during wave 3. Furthermore, the range of CFR values observed during wave 1 was much wider than for later waves.

**Table 2:** Observed, age-standarized cumulative incidence (per 10,000 population) and case fatality rates (CFRs; %) for the first five waves of COVID-19 in Germany, as well as the extent of observed spatial clustering, as measured by Moran’s I

Pandemic Wave		Incidence (per 10,000 population)		CFR (%)	
		Median (Range)	Moran’s I (95% CI)	Median (Range)	Moran’s I (95% CI)
1	1.1	9.56 (1.60, 103.2)	0.58 (0.52, 0.64)	3.45 (0, 19.0)	0.084 (0.019, 0.15)
	1.2	7.33 (0.45, 52.1)	0.56 (0.49, 0.62)	5.00 (0, 16.7)	0.070 (0.005, 0.14)
2		243.0 (57.8, 640.6)	0.69 (0.63, 0.76)	3.06 (0.74, 6.77)	0.19 (0.13, 0.26)
3		141.6 (37.7, 457.2)	0.62 (0.56, 0.69)	0.67 (0.08, 2.67)	0.34 (0.27, 0.40)
4		365.0 (110.1, 1296)	0.87 (0.80, 0.93)	1.05 (0, 4.41)	0.53 (0.46, 0.59)
5		2364 (1424, 3387)	0.65 (0.58, 0.71)	1.12 (0.14, 5.85)	0.36 (0.29, 0.42)

Both incidence and CFR were significantly clustered in space, but incidence showed much stronger clustering than CFR. For both incidence and CFR, clustering was highest during wave 4 (Table 2). Cumulative incidence for a given wave was typically positively correlated with the cumulative incidence in other waves, indicating that relative incidence was fairly conserved over time. For CFR, a similar pattern was observed for waves 3 through 5, but not for the earliest waves.

### Incidence model

Including demographic and socioeconomic predictors significantly improved model fit over null models in all waves, as assessed using AIC, although the magnitude of this improvement varied by wave. Models including all predictors explained 75.4%, 70.4%, 80.2%, 85.0%, 93.3%, and 75.0% of deviance in waves 1.1, 1.2, 2, 3, 4, and 5, respectively. In comparison, null models explained 72.7%, 69.0%, 73.9%, 78.7%, 92.2%, and 68.7% of deviance for each wave.

**Fig. 1:**
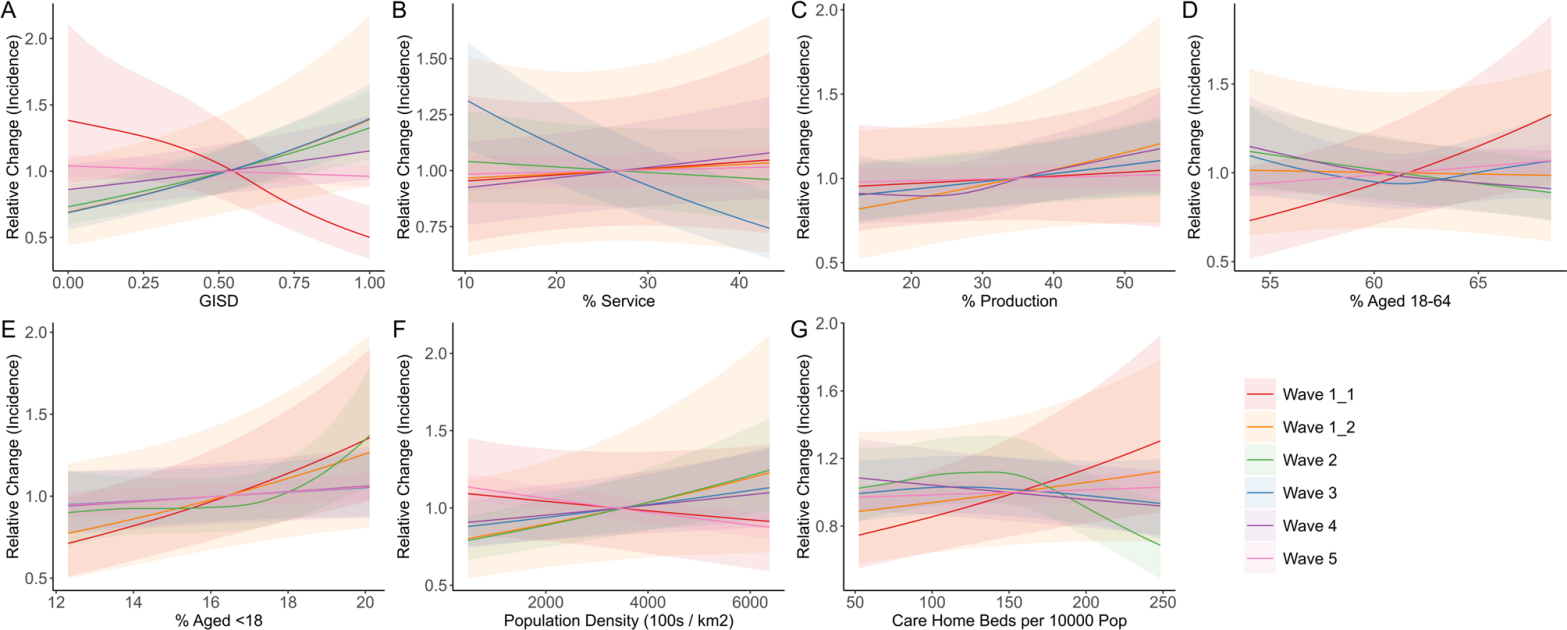
Predicted multiplicative change in COVID-19 incidence with changing values of district-level demographic, socioeconomic, and healthcare variables, by pandemic wave. Solid lines represent the marginal effect of each predictor variable when all other predictors are set to their mean values; values above 1.0 indicate increased predicted incidence relative to the mean impact of the variable of interest, while values below 1.0 indicate decreased incidence. Shaded areas represent 95% confidence intervals. (**A**) German Index of Socioeconomic Deprivation; (**B**) percent of workers in person-related service jobs; (**C**) percent of workers in production-oriented jobs; (**D**) percent of the population aged 18–64; (**E**) percent of the population aged less than 18; (**F**) population density in 100 s of people per square kilometer; (**G**) spots in long-term care facilities per 10,000 population

The fitted associations between the demographic, healthcare, and socioeconomic predictor variables and district-level COVID-19 incidence can be seen in Fig. 1. As expected based on previous studies, the relationship between socioeconomic deprivation and COVID-19 incidence changed over time (Fig. 1a). During the first part of wave 1 of the pandemic, increased deprivation is associated with reduced incidence relative to the mean. Beginning in the second part of wave 1, the opposite relationship is observed, with incidence increasing fairly linearly with deprivation. By wave 4, this relationship has substantially weakened, and in wave 5, a clear relationship between deprivation and incidence no longer exists. Unlike the GISD score, neither the percentage of workers in service jobs nor in production jobs is strongly associated with incidence in any wave, with the exception of a decrease in incidence with an increasing proportion of service workers in wave 3 (Fig. 1b-c).

**Fig. 2:**
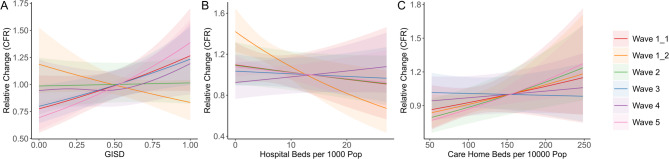
Predicted multiplicative change in COVID-19 case fatality rates with changing values of district-level socioeconomic and healthcare variables, by pandemic wave. Solid lines represent the marginal effect of each predictor variable when all other predictors are set to their mean values; values above 1.0 indicate increased predicted incidence relative to the mean impact of the variable of interest, while values below 1.0 indicate decreased incidence. Shaded areas represent 95% confidence intervals. (**A**) German Index of Socioeconomic Deprivation; (**B**) hospital beds per 1000 population; (**C**) spots in long-term care facilities per 10,000 population

I understand if this is not possible at this point, but just in case, I have uploaded new versions of Figures 1 and 2, where the legend is only included once (rather than being repeated in each panel). None of the results being plotted have changed.The proportion of a population who are children is more consistently related to incidence than the proportion of working-age adults overall (Fig. 1d-e). In waves 1 and 2 in particular, districts with more children tended to experience higher incidence, while the proportion of working-age adults was only associated with a notable increase in incidence during wave 1.1. Population density was only weakly associated with incidence in all waves (Fig. 1f). Districts with the greatest number of care home beds per capita experienced higher incidence in wave 1.1, but lower incidence in wave 2; in all other waves, there was little association between care home beds and incidence (Fig. 1g).

### CFR model

Including demographic, healthcare, and socioeconomic predictors significantly improved models of CFR for all waves except wave 3, with the greatest improvements during later waves. Models of CFR explained a much lower percentage of total deviance than models of incidence (by wave, 17.7%, 15.2%, 34.4%, 35.9%, 32.4%, and 15.1%). Here, null models explained 19.7%, 14.6%, 31.7%, 35.3%, 28.6%, and 10.2% of deviance for each wave.

Model-inferred associations between CFR and the included socioeconomic and healthcare predictors are shown in Fig. 2. Early in the first pandemic wave, we find evidence of higher CFRs in more deprived districts. While this association reverses in direction later in the wave, it reappears by wave 3, and the strongest relationship between district-level deprivation and CFRs was observed in wave 5 (Fig. 2a). During wave 3, the strength of the association increased with decreasing population density, and a negative association was observed when population density was high (Figure S6). During wave 1.2 only, an increase in hospital beds per capita was associated with lower CFRs (Fig. 2b). An increase in care home beds per capita was associated with a slight increase in CFR in wave 1, and a stronger increase in CFR in waves 2 and 5; in waves 3 and 4, little association was observed (Fig. 2c).

### Impact of incidence on CFR

**Fig. 3:**
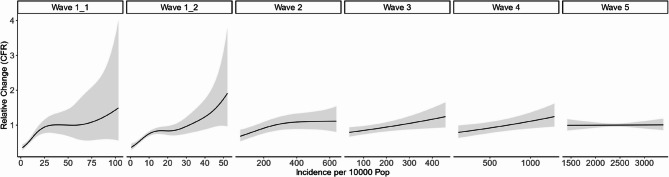
Predicted multiplicative change in COVID-19 case fatality rates as incidence per 10,000 population varies, by wave. Solid lines represent the marginal effect of incidence when all other predictors are held constant at their mean values, while shaded areas represent 95% confidence intervals. Note that, because of variation in incidence rates by wave, the scale of the x-axis is different in each wave

Across waves 1 through 4, an increase in wave-specific COVID-19 incidence was associated with an increase in CFR, even after including district-specific cases as an offset (Fig. 3). This relationship was particularly strong in wave 1, where we see a rapid increase in CFR as incidence increases, especially at incidence rates below about 40 cases per 10,000 population in wave 1.1 and about 16 cases per 10,000 population in wave 1.2. We note that, as the majority of districts experienced incidence rates below these values during wave 1, statistical support for the association at higher incidence values is weaker, as reflected by the relatively wide confidence intervals. In contrast, during wave 5, no relationship between incidence and CFR was observed. These results suggest that, during the first four waves of the pandemic, deaths did not scale linearly with cases, but rather that cases were more likely to lead to death when incidence was higher.

### Spatial analyses

Partial Mantel correlation coefficients comparing commuting flows and geographic distances to the underlying spatial patterns in COVID-19 incidence, after controlling for all other variables, can be found in Table 3. Note that, because higher values in the commuting matrix denote larger commuting flows between districts rather than larger distances, negative values for partial Mantel tests of spatial distribution and commuting flows reflect a greater similarity between districts with higher rates of between-district commuting.

**Table 3:** Associations between COVID-19 incidence and commuting, controlling for geographic distance, and between COVID-19 incidence and geographic distance, controlling for commuting, during the first five pandemic waves in Germany

Pandemic Wave		Partial Mantel Correlation	
		Incidence vs. Commuting	Incidence vs. Distance
1	1.1	−0.062*	0.329*
	1.2	−0.209**	0.106*
2		−0.105**	0.364*
3		−0.211**	0.122*
4		0.002	0.515*
5		−0.048	0.120*

*p < 0.01, **p < 0.001

We find that, after controlling for geographic distance, incidence patterns are only significantly associated with commuting flows in waves 1.2 through 3 (using a Bonferroni-adjusted cutoff for significance of *p* = 0.002); in waves 1.2 and 3, this relationship was stronger than that between incidence and geographic distance, controlling for commuting flows.

## Discussion

In this study, we assessed how district-level COVID-19 incidence and CFRs varied according to a variety of socioeconomic, demographic, and healthcare-related predictors over the first five pandemic waves in Germany. We found evidence of socioeconomic inequalities in both incidence and CFRs that persisted across multiple pandemic waves. More specifically, while associations with incidence waned over the course of the pandemic, associations with CFR appeared most pronounced during later waves, possibly indicating increasing socioeconomic disparities. Additionally, we show that deaths due to COVID-19 do not appear to scale linearly with cases, such that increases in incidence are associated with increased case fatality rates, particularly during earlier waves.

Consistent with previous results in Germany [18, 19], our results suggest that incidence was higher in less deprived regions at the beginning of the outbreak, likely due to introductions by wealthier residents returning from international vacations [19]. However, the direction of this relationship reversed within six weeks, and more deprived regions experienced higher incidence throughout waves 2 and 3. There are several plausible reasons for this relationship. In particular, individuals with lower SEP are less likely to have jobs that can be performed remotely, and are therefore less likely to be able to social distance effectively [43]. Indeed, further studies in Germany have shown that those with lower educational attainment and occupational status were less likely to reduce their contacts, at least during the first pandemic wave [57], and that the ability to work from home was in part responsible for the link between educational attainment and risk of infection [23]. If workers with higher SEP tended to return to in-person work once permitted, this may also explain why the association between deprivation and incidence seemed to fade in waves 4 and 5, when many control strategies had been relaxed or removed. Notably, a serological study in Germany found that undetected infections with SARS-CoV-2 were more common among those with lower levels of educational attainment [25, 58], suggesting that the true relationship between deprivation and incidence could be even stronger than our results would indicate, and may even persist at a weaker level into waves 4 and 5.

The patterns we observed early in the pandemic, in which incidence is initially higher in less-deprived regions before rapidly concentrating in more-deprived regions, are not unique to Germany: in Hong Kong, high incidence rates early in the pandemic were also observed among wealthy individuals traveling internationally [6], and a systematic review found similar patterns in several other countries as well [9]. Our finding that socioeconomic inequalities in incidence waned as the pandemic progressed was seen less often in other contexts, although we note that few studies have covered time periods as long as that captured here.

Socioeconomic inequalities were also evident in CFRs, although the strengths of these associations were typically weaker than those observed between deprivation and incidence. Unlike with incidence, we found that the strength of the association between deprivation and CFR increased from wave 2 through wave 5. These inequalities could be due to the fact that individuals with lower SEP might have had less access to high-quality medical care [59, 60] as well as higher rates of chronic health conditions such as hypertension and diabetes that exacerbate COVID-19 severity [61]. As noted above, a German serological study suggested that underestimates of incidence may be particularly pronounced among those with lower educational attainment. Because this may artificially inflate CFRs in more-deprived regions, it is possible that the true strength of these associations is somewhat lower than observed here. Depending on how socioeconomic disparities in testing evolved over time, potentially in response to factors such as testing requirements for accessing public spaces or using public transport, the difference between the true association strength and that estimated here may also vary over time. That said, our finding that inequalities appear to have increased over time deserves further attention. In particular, future work should assess whether such inequalities are also present at the individual level, and, if so, how individuals with low SEP can be better protected.

Because socioeconomic deprivation contributes broadly to morbidity and mortality [61], it is difficult to judge whether the observed associations are reflective of general trends, or if associations between socioeconomic deprivation and COVID-19-specific outcomes were particularly strong. Studies of life expectancy in Germany have found that socioeconomic disparities increased especially rapidly during the pandemic [62, 63], and research from the United States showed that associations between various socioeconomic predictors and all-cause mortality were more volatile during the COVID-19 pandemic than in the pre-pandemic period [64]. Because our work focuses on COVID-19-specific outcomes (incidence and CFRs) that cannot be calculated prior to SARS-CoV-2 emergence, such a direct comparison is not possible here. Nonetheless, we conducted a sensitivity analysis in which we fit models of district-level all-cause mortality during the five years leading up to the pandemic (see Supplementary Materials). As in [64], we find that the association between the GISD and all-cause mortality is highly stable during the pre-pandemic period. This could indicate a role for pandemic-specific factors (e.g., differential ability to comply with social distancing measures [23, 57]) in driving the associations identified here.

In addition to socioeconomic deprivation, we explored the relationships between COVID-19 impact and several potential explanatory variables. As with socioeconomic deprivation, the strength and sometimes direction of these relationships varied between waves. For example, a higher number of hospital beds per capita was associated with reduced CFRs in wave 1, but not in subsequent waves. This may indicate that regions with more hospital beds were less prone to hospital overcrowding, which could have had a particularly large impact on mortality during the first wave, when it was still uncertain how best to treat patients with severe COVID-19. Alternatively, a greater number of hospital beds may be correlated with an increased capacity for specialized therapies such as extracorporeal membrane oxygenation (ECMO), although we note that mortality rates remained high among severe cases receiving such treatments [65]. Interestingly, we found no consistent relationship between the percentage of workers in service or production jobs and COVID-19 incidence, despite the fact that these jobs have been associated with a higher risk of SARS-CoV-2 infection and mortality in previous work, at least during some waves [24, 66–69]. It is possible that this is due to the ecological nature of our study, which only allows us to compare district-level incidence according to the percentage of individuals working in different sectors. Individual-level studies, which would allow for direct comparisons between individuals working within and outside of these sectors, may reveal more substantial associations. The lack of an identified association between incidence and population density is also surprising [70, 71], but could be due to a similar reason. Namely, district-level population density may be less relevant than, for example, individual-level differences in household sizes [5].

A particularly striking finding of our study was the tendency for districts with higher incidence to also experience higher CFRs, especially in the early waves. In other words, the total number of deaths does not scale linearly with the total number of cases. A similar pattern was previously observed during the early stages of the first wave in Italy [72]. Because infectious disease surveillance data often underestimate the true number of cases and deaths, it is important to consider up front whether this finding could be, at least in part, due to inaccuracies in data collection. For example, if patients with mild illness are more likely to forgo examination and testing when medical and testing facilities are overcrowded, CFRs may appear to increase more rapidly than incidence rates. Regions with better-quality healthcare may also be better able to correctly identify both cases and deaths due to COVID-19, potentially biasing the association between incidence and CFRs. Because healthcare quality likely also influences reported case counts (i.e., it affects both the numerator and denominator when calculating CFR), the direction of this bias is unclear.

With that said, there are a number of possible mechanisms by which increased incidence could have directly contributed to higher CFRs. First, it is possible that higher incidence led to an increased burden on the healthcare system, which could have compromised quality of care. This would be expected to play a particularly large role in early waves, and a much smaller role in later waves, when high vaccination rates, a younger average age of infection, and the emergence of milder viral variants greatly reduced CFRs regardless of healthcare factors. Notably, this relationship exists despite the fact that the majority of intensive care units in Germany reported normal operating conditions during the first wave [73], perhaps suggesting that patient care may begin to be impacted even at low levels of crowding and staff shortages. It is important that future work explore this further. It is also possible that, as overall incidence increased, the probability that groups most at risk of death, including the elderly and individuals with chronic health conditions, would escape infection decreased, leading to overall greater CFRs. Finally, there is evidence suggesting that those initially exposed to a higher dose of a virus tend to experience more severe disease [50, 74, 75]. Regions with higher incidence will also have higher force of infection, which could be a proxy for infectious dose. If so, new cases in regions with higher incidence may be exposed to a higher initial viral load, which could in turn increase their risk of severe disease. Alternatively, higher incidence regions, particularly early in the pandemic, could be associated with more superspreading events, in which those transmitting the infection may have higher viral loads [76]. Additional research is needed to clarify the extent to which force of infection affects disease severity, as well as whether this played a role in exacerbating CFRs due to COVID-19.

Our spatial analysis suggests that, in most waves, commuting was not a primary driver of the observed spatial patterns in COVID-19 incidence. This may be in part due to the increase in employees working from home during the pandemic [77]. Interestingly, commuting was associated with incidence patterns during wave 3, despite the fact that regulations at that time required employers to allow employees to work from home if possible [78]. Meanwhile, during the fourth and fifth waves, many cases occurred among those younger than 18, rather than working-age adults, potentially explaining the lack of a role for commuting later in the pandemic.

While our work has expanded on past research in a number of important ways, we are cognizant of several limitations. In particular, the potential biases associated with the use of surveillance data must be adequately considered. Although COVID-19 is a reportable disease in Germany, only laboratory-confirmed cases are included in the data, and many mild and asymptomatic cases, which make up a large portion of SARS-CoV-2 infections [79], are therefore likely to be missed. As described above, a serological study found that, in Germany, underreporting was especially pronounced among those with lower levels of formal education [25, 58]. For this reason, it is possible that the associations we detected between socioeconomic deprivation and COVID-19 incidence were biased downward, while the associations we found with CFRs were overestimated. While we cannot say with certainty how the extent of this bias may have changed over time, it is possible that bias was particularly high during wave 1, before testing became more routine, and during wave 5, when the emergence of the Omicron variant led to particularly high incidence in combination with an overall milder clinical course [80].

Our mortality data contains all deaths associated with a positive SARS-CoV-2 test, and not only deaths judged to be due to SARS-CoV-2 infection specifically, which should at least partially mitigate the risk of underreporting. Evidence from the first three pandemic waves suggests that the majority (> 80%) of these patients had COVID-19 as an underlying cause of death, and not merely as an associated condition [81, 82]. However, just as with the incidence data, we cannot rule out the possibility that underreporting varies according to our predictors of interest. Indeed, some research suggests that COVID-19 deaths may be particularly underestimated in disadvantaged and in nonmetropolitan regions [83–85]. Ideally, we would refit our models of CFR using excess deaths, rather than reported COVID-19 deaths, as a sensitivity analysis to assess the extent to which differences in underreporting may have influenced our results. By comparing observed all-cause mortality during the pandemic to the mortality expected based on existing trends, excess death data do not require the correct diagnosis of all patients dying of COVID-19, and are therefore less affected by regional differences in, for example, testing capacity or healthcare quality [86, 87]. Unfortunately, due to a lack of weekly all-cause mortality data at the district level, we were unable to do this analysis. However, we were able to calculate wave-specific excess deaths at the state level, and to compare these with reported COVID-19 deaths. Encouragingly, we found that the number of state-specific excess and reported deaths were highly correlated in Germany, suggesting a lack of spatial bias in the extent of underreporting (see Supplementary Materials). We also note that an analysis comparing excess and COVID-19 specific deaths in several countries found that COVID-19-specific deaths typically equaled or exceeded excess deaths in Germany, suggesting that substantial underreporting of deaths was uncommon [87].

As a further limitation, this is an ecological study; while we are able to say that districts with certain characteristics experience higher or lower incidence or CFRs, we are unable to draw any conclusions about associations at the level of the individual [88]. Furthermore, by aggregating over a relatively large spatial scale, we ignore the substantial heterogeneity between individuals within a district, potentially obscuring associations that operate at smaller spatial scales or at the individual level. Bias may also occur due to the fact that decisions concerning where to draw borders between districts are statistically arbitrary (the modifiable areal unit problem [89]).

We also did not control for differences in adherence to COVID-19 control measures, or for rates of underlying health conditions known to exacerbate the severity of COVID-19, mainly due to a lack of high-quality and freely-available data. These are both potentially important mediators of the association between deprivation and COVID-19 impact, and including them in the final models could therefore identify potential, hypothesized mechanisms by which socioeconomic deprivation may lead to higher COVID-19 incidence and CFRs. In later waves, we controlled for vaccination rates, which have been shown to be lower for those with lower income or lower educational attainment in Germany [90].

Finally, due to data limitations, we were unable to consider the extent to which COVID-19 burden was associated with immigration status and ethnicity. While data on the proportion of a district’s population who are immigrants exist, more granular data, including information on region of origin or proficiency with German or English, do not, nor do data on race or ethnicity. Both immigrants and people of color may experience difficulties accessing medical care due to factors like language barriers and discrimination from healthcare providers, which may in turn put them at greater risk for severe disease outcomes, including death [91]. Race and ethnicity have been reported as important drivers of COVID-19 burden in other countries [3, 92], and early results from Germany suggest that immigrants, children of immigrants, and those speaking a language other than German at home were more likely to be infected with SARS-CoV-2 [93]. For this reason, it is important that future individual-level studies consider these factors, if possible.

Our work has demonstrated the existence of socioeconomic inequalities in COVID-19 incidence and CFRs at the district level, and it is crucial that public health practitioners take such inequalities into account when planning and implementing control measures. Failure to do so may result in control strategies that do not adequately protect those who are most vulnerable. Future research should consider the extent to which different control strategies were more or less effective in populations with different SEP. Furthermore, it is likely that non-COVID health conditions were also exacerbated by the pandemic, due to both healthcare disruption [94] as well as increases in economic hardship and stress [95, 96]. Future work should also assess the extent to which non-COVID-19 disease burden and mortality differs by SEP. Insights gained from such work will be useful not only in future waves of COVID-19, but also in the case of future pandemics involving respiratory infections.

## Supplementary Information

Supplementary Material 1.

## Data Availability

Data on district-level numbers of COVID-19 cases and deaths, as well as population sizes, are available from [infas 360](https:/datenkatalog.infas360.de) (under “Datensätze,” then “Corona-Datenplattform”). To access the data, an account must first be made; accounts can be requested by individuals with a relevant affiliation. The German Index of Socioeconomic Deprivation data can be downloaded [here](https:/github.com/robert-koch-institut/German_Index_of_Socioeconomic_Deprivation_GISD); data on all other predictor variables are available from [INKAR](https:/www.inkar.de). Finally, code to obtain estimates of regional vaccination rates as well as between-district commuting data can be found [here](https:/github.com/SciCompMod/memilio/tree/main). All code used in the analyses described in this manuscript, including code to format the raw data, is freely available on Github: https://github.com/sarahckramer/covid_ses.

## Abbreviations

AIC: Akaike information criterion
BIC: Bayesian information criterion
CFR: Case fatality rate
COVID-19: Coronavirus disease 2019
DHARMa: Diagnostics for HierArchical Regression Models
ECMO: Extracorporeal membrane oxygenation
GAM: Generalized additive model
GISD: German Index of Socioeconomic Deprivation
GLM: Generalized linear model
PCR: Polymerase chain reaction
SARS-CoV-2: Severe acute respiratory syndrome coronavirus 2
SEP: Socioeconomic position
