# Supplementary material for: Changing socioeconomic inequalities in the incidence and case fatality rates of COVID-19 in Germany, March 2020 through May 2022: an ecological study
Source: BMC Public Health. 2025 Oct 2;25:3289. doi: 10.1186/s12889-025-24625-9 (PMC12492518; doi:10.1186/s12889-025-24625-9)
Supplement: Supplementary file 1 — Supplementary Material 1. [file 12889_2025_24625_MOESM1_ESM.pdf]

Timeline of the COVID-19 Pandemic in Germany

Government Response

A plot of the stringency index, a composite measure including information on the intensity of country-level measures to contain COVID-19 (1), over the course of the study period can be found in Figure S2A. The time periods of major, country-level control measures are also displayed. We note that, although there were several country-level control measures in place throughout the majority of the study period, the exact range of measures taken, as well as the precise timing of implementation, varied by state. Nonetheless, we have tried to highlight the general state of nonpharmaceutical interventions over time throughout Germany.

Germany experienced two major “lockdowns” during the early pandemic, the first beginning on 22 March 2020, and the second beginning on 16 December 2020 (2,3). Lockdowns included widespread closure of workplaces, shops, and schools; restrictions on contacts outside of one’s household; and physical distancing in public spaces. Prior to the second major lockdown, there was also a short “lockdown light” beginning on 2 November, during which social distancing measures were less stringent, and schools and daycare centers remained open. Each major lockdown was phased out gradually, starting from 20 April 2020 for the first and from 3 March 2021 for the second (2). Throughout the pandemic, Germany also instituted a so-called “Hotspot-Strategy,” which enforced a temporary return to more stringent contact restrictions in individual districts whenever the 7-day incidence exceeded a certain threshold (typically between 35 to 50 cases per 100,000 residents) (4,5). Even after the lockdowns ended, employers were obligated to allow employees to work from home if possible; this policy remained in place through June 2021 (6) and was reinstated in November 2021 (7).

Beginning in August 2021, so-called “3G-Rules” were implemented, which allowed participation in many aspects of public life for those who were fully vaccinated (“geimpft”), had received a negative test within the past 24 hours (“getestet”), or who could prove recovery from a SARS-CoV-2 infection (“genesen”) (8). Both PCR and rapid tests were permitted, and, throughout much of our study period, rapid tests could be obtained free of charge by at widely distributed test centers. Certain events or locations instead employed the stricter 2G (only vaccinated and recovered) or 2G+ (vaccinated or recovered, plus a negative test) rules (9).

Finally, mask mandates in certain public spaces went into effect in all 16 states between 20 and 29 April 2020 (10), although whether penalties existed for noncompliance varied by state. Beginning in late January 2021, cloth masks were no longer considered sufficient, and medical-grade masks (such as FFP2 or surgical masks) specifically were required (11). Mask mandates broadly remained in place even after lockdown periods ended, although again, this varied geographically.

Almost all country-level control measures ended on 3 April 2022; one major exception was the continuation of mask mandates in certain contexts, including in hospitals and on public transportation (12,13).

Testing

The number of tests performed for SARS-CoV-2 in Germany over the course of the pandemic is plotted in Figure S2B. As stated in the main text, testing of suspected COVID-19 cases by healthcare providers was encouraged based both on symptoms and on contact history (see “COVID-19 Data”), although the exact recommendations varied over the course of the pandemic. Broadly, guidelines concerning who to test for SARS-CoV-2 were stricter during the first two waves, where testing was often limited to those with severe symptoms, belonging to high risk groups, or with recent contact with someone confirmed to be infected with SARS-CoV-2 (14).

In addition to testing by healthcare providers, PCR tests were available to the general public throughout much of the pandemic at test centers. During some stages of the pandemic, PCR tests were free for those at high risk of infection or with a positive rapid test (15). However, because a negative result from a rapid antigen test was sufficient to allow participation in most activities under 3G-Rules (see above) (8), it is unlikely that the majority of infected individuals accessed PCR tests specifically.

Vaccination

The percentage of the German population fully vaccinated (defined below under “Defining the proportion vaccinated against SARS-CoV-2”) against SARS-CoV-2 is shown in Figure S2C. German residents were classified into four major priority groups, which determined when each resident was eligible for vaccination (16). Broadly, these groups were:

Group 1: Those >80 years old, residents and staff of long-term care facilities, medical staff working in high-risk environments
Group 2: Those >70 years old, those with a very high risk of severe SARS-CoV-2 infection (due to various chronic conditions), medical staff
Group 3: Those >60 years old, those with an increased risk of severe SARS-CoV-2 infection, essential workers
Group 4: All remaining residents aged 16 and above

Vaccines were first made available on 26 December 2020 for the highest priority group (17), and access was expanded over time to each of the next priority groups in turn, although the exact dates varied by state. On 7 June 2021, the prioritization period ended (18), and access was further expanded to children aged 12-15 (19). Beginning on 13 December 2021, vaccination was permitted in children aged 5 and above (20).

By the end of our study period, 76.2% of the German population had been fully vaccinated against SARS-CoV-2. In general, coverage levels were similar across districts (Figure S2C), although particularly low vaccination rates were observed in Saxony (all 13 districts of Saxony had coverage rates of less than 70% during wave 5, compared to a mean coverage of 75.1% across all districts of Germany) and in the southernmost regions of Baden-Württemberg.

Supplementary Methods

Defining Pandemic Waves

Given the high degree of variability in outbreak shape and size by wave (Figure S1), we opted to explore a variety of metrics when defining individual waves, rather than applying a strict definition to all waves. Where possible, we chose start and end dates consistent with choices made in the literature. This was done to maximize comparability between our study and previous work. A descriptive study written by several employees of the Robert Koch Institute (RKI), the German government's central biomedical and public health institute, defined wave 1 as including weeks 10 through 20 of 2020 and wave 2 as including week 40 of 2020 through week 8 of 2021 (21). This cutoff for wave 1 is in agreement with an article exploring the socioeconomic correlates of COVID-19 incidence and mortality rates during the first wave, which defined week 20, when existing lockdown measures were relaxed, as the end of the wave (22). Meanwhile, wave 2 was defined similarly in (23). Because the data used for this study began in week 9 of 2020, we included this week in the first wave, and defined wave 1 as weeks 9 through 20; wave 2 was defined as in (21).

Because fewer studies have been conducted using data from later waves, wave start and end dates were rarely available. We therefore chose cutoffs for the three later waves based on epidemic activity and characteristics at the country level. As incidence began increasing immediately after the end of wave 2, we defined wave 3 as beginning in week 9 of 2021. Wave 3 continued through week 21 of 2021, the first week during which country-level incidence fell below 50 cases per 100,000 population. The beginning of wave 4 was defined similarly, as the week prior to the first week in which country-level incidence again exceeded 50. In agreement with reports published by the RKI, we defined the start of wave 5 (and therefore the end of wave 4) as week 52 of 2021, the first week in which the Omicron variant represented the majority of sampled infections (24). We chose week 21 of 2022 as the end of wave 5 because the country-level incidence reached a trough at this time point, before rapidly increasing again to another peak.

Defining Partial Pandemic Waves

As described in the main text, wave 1 of the pandemic was divided into two partial waves for all analyses. We tested cutoffs at weeks 10 through 18, and fit models of COVID-19 incidence including all predictors of interest for the two partial waves as defined by each potential cutoff week. The highest total deviance explained for the two partial waves combined occurred with a cutoff at the end of week 14. We also plotted the marginal effect of SEP on incidence for the two partial waves for all cutoff weeks, and found that the relationship between GISD and COVID-19 incidence differed most noticeably between the two partial waves with a cutoff at the end of week 14, suggesting that this cutoff also represents the timepoint at which the change in the association between SEP and incidence is most prominent. This choice of cutoff was not sensitive to the chosen value of k for the spatial smooths. Our choice of cutoff week is consistent with (22), who suggested a cutoff around one week after the beginning of the first lockdown period at the end of week 12, and who found opposite associations with district-level measures of SEP when comparing incidence and mortality before and after this cutoff.

Additionally, we conducted sensitivity analyses where waves 2 through 5 were also split into partial waves. For wave 2, we defined the first partial wave as continuing through week 48. A descriptive analysis of the second wave found that incidence became highest in districts with GISD in the top quintile beginning in week 49 (25). Thus, this choice of cutoff allows us to evaluate whether we also find evidence of a change in the direction of the association between SEP and incidence at this timepoint, when controlling for spatial dependence and other covariates. For wave 3, the cutoff was placed at the wave's peak; for waves 4 and 5, which each contained two distinct peaks, cutoffs were chosen to be the troughs between the two peaks. Weeks included in each partial wave for waves 2 through 5 can be found in Table S1 below.

**Table S1.** Partial wave definitions for the first five waves of the COVID-19 pandemic in Germany. The end dates for each week are included in parentheses.		
Wave 1	week 9, 2020 (Mar 1) – week 14, 2020 (Apr 5)	week 15, 2020 (Apr 12) – week 20, 2020 (May 17)
Wave 2	week 40, 2020 (Oct 4) – week 48, 2020 (Nov 29)	week 49, 2020 (Dec 6) – week 8, 2021 (Feb 28)
Wave 3	week 9, 2021 (Mar 7) – week 16, 2021 (Apr 25)	week 17, 2021 (May 2) – week 21, 2021 (May 30)
Wave 4	week 32, 2021 (Aug 15) – week 40, 2021 (Oct 10)	week 41, 2021 (Oct 17) – week 51, 2021 (Dec 26)
Wave 5	week 52, 2021 (Jan 2) – week 8, 2022 (Feb 27)	week 9, 2022 (Mar 6) – week 21, 2022 (May 29)

Age Standardization

As stated in the main text, COVID-19 data were stratified into six age groups: 0-4, 5-14, 15-34, 35-59, 60-79, and 80+; these age groups were therefore also used for the age standardization. Population data from 2020, also retrieved from the Corona Data Platform, were used to obtain the reference age distribution for the entire country. Age-standardized incidence and mortality rates were calculated as:

$$X_{stand} = \sum_{A} X_a \left(\frac{N_a^{ref}}{\sum_A N_a^{ref}} \right)$$

where X_{stand} is the age-standardized incidence or mortality rate, X_a is the raw incidence or mortality rate in age group *a* in the population being studied, N_a^{ref} is the number of people in the reference population belonging to age group *a*, and *A* indicates all age groups in the population (26). The age-standardized number of cases or deaths for each district can then be calculated by multiplying the age-standardized incidence or mortality rate for each district by the district's total population size, and rounding to the nearest whole number.

Occasionally, the data contain cases and deaths without age information; when this happened, the data were assigned an age group based on the relative proportion of cases or deaths in each age group that day. If a given date contained only cases or deaths with no age information, these data were removed from the dataset. The latter scenario happened very infrequently: only eleven such cases and six such deaths were identified.

Additionally, we found that the county of Heinsberg was lacking data on 57 cases in the incident relative to the cumulative data. This is most likely due to the fact that Heinsberg was one of the first counties in Germany to be hit by COVID-19, and these 57 cases occurred before March 1, 2020, the first date for which incident data are available. To account for this, we distributed these 57 cases among the six age groups proportionally to the age distribution of cases on March 1, 2020.

German Index of Socioeconomic Deprivation (GISD)

The GISD was developed by the RKI in 2017, and updated in 2022, to describe the relative extent of socioeconomic deprivation in Germany at various levels of spatial aggregation (27). Initially, the authors searched the INKAR database (28) for indicators related to three key "dimensions" of socioeconomic deprivation: education, employment, and income. The following indicators were chosen for each dimension:

- Education: Proportion of employees with a university degree; proportion of employees with no formal professional qualifications; proportion of school leavers without a lower secondary school certificate
- Employment: Unemployment rate; employment rate; monthly gross salary per employed inhabitant
- Income: Average net household income per inhabitant; private debtors per 100 inhabitants; income tax per inhabitant

These indicators were chosen based on a systematic review of the literature, as well as for their availability at relatively small spatial scales and over several years. Where data were missing, they were imputed using linear random intercept models. Additionally, the values of several indicators were adjusted in at least some years to account for various statistical and historical artifacts (see (27) for more details).

Next, principal component analyses were used to determine the indicator-specific weighting for each of the three dimensions, using data from 2001 through 2019. Yearly scores for each dimension were normalized to vary between 0 and 1. Finally, GISD scores were calculated by combining the scores for the three dimensions, such that each dimension was weighted equally (33.3%). Scores at the municipality level were aggregated to the district level by weighting according to population size. Thus, the district-level GISD scores range from 0 (for the least deprived district) to 1 (for the most deprived district). The distribution of GISD scores across Germany is shown in Figure S3; although we plot the scores by quintile, our models make use of the raw GISD score for each district. For additional details on the conception and construction of the GISD, see (27) and (29).

Additional Model-Fitting Details

Defining the proportion vaccinated against SARS-CoV-2

Because vaccines against SARS-CoV-2 take about two weeks to reach full effectiveness (30,31), models including vaccination rates consider the cumulative proportion of the population vaccinated two weeks prior to a wave's midpoint. Specifically, we include the proportion fully vaccinated, defined as those who had either 1) received two doses of any SARS-CoV-2 vaccine , 2) received a single dose of the Janssen vaccine, or 3) received a single dose of any SARS-CoV-2 vaccine and also recovered from infection with SARS-CoV-2 (32).

Modeling the spatial smooth

For the majority of districts, we modeled the spatial smooth using the latitude and longitude of each district's centroid. For a small number of districts (n = 19), centroids were located outside of the district itself; in these cases, we instead used the "st_point_on_surface" function from the R package sf (version 1.0.16) (33,34), which attempts to find a central point guaranteed to be located within a given polygon, and assigned the district the latitude and longitude of the resulting point. Spatial smooths were all modeled using Duchon splines with first derivative penalization, which have relatively low sensitivity to values along the boundary of a space (35). We tested several alternative spatial smooth types, including splines on the sphere and Gaussian process smooths, but these neither consistently improved fit nor changed effect estimates.

As stated in the main text, appropriate choices of the basis dimension ("k") for the spatial smooths was particularly important. Broadly, decisions regarding these values were chosen based on the AIC and BIC of models fit with a range of k-values. However, we note that there was often a range of values yielding models with similar AIC and BIC. In these cases, specific values of k were chosen such that the value was high enough to prevent any residual spatial autocorrelation (as evaluated by calculating Moran's I for the model residuals), but low enough to prevent substantial overfitting (as tested using the DHARMa package (version 0.4.6) (36)). Notably, the k-value acts as an upper bound on the complexity of the modeled association; if the maximum amount of complexity is not needed to fit the data, the fitted GAM may return a smooth with less complexity than permitted by k. For this reason, it is not unusual to find that a range of values will yield similar model fit. Where possible, lower values were chosen in order to minimize computation times.

Modeling smooths for predictors of interest

For all independent variables other than the spatial smooth and the state-level random effect, k-values were set to 10, the default value used by the mgcv package (version 1.9.1) (35). Fitted models were then checked using the "gam.check" function, which tests whether there are remaining patterns in the residuals according to each predictor, and k-values were increased arbitrarily to 25 if potential issues were found. Smooths for all predictors except the spatial smooth and the state-level random effects were modeled using thin plate regression splines, which are highly flexible, and do not require knot locations to be specified (37). Thin plate regression splines are the default smooth type used by mgcv. Interaction terms were modeled using tensor product interactions, which allow for differences in the scales of the interacting variables (35).

Model selection and comparison

All models were fit using maximum likelihood methods, to allow for statistical comparisons between models using different predictors. For all models presented in the main text, we also tested models 1) including average living area per person as an additional predictor, and 2) modeling outcomes using a Tweedie distribution, an alternative approach when Poisson models yield evidence of overdispersion. We then compared the fit of these models to those in the main text using BIC values. Neither inclusion of living area as a predictor nor use of a Tweedie distribution consistently improved model fit across waves and outcome measures.

We tested interactions for inclusion by fitting models with no interactions, and comparing their BIC values to those of models including all potential interactions of interest. For models of incidence, interactions of interest were between GISD and age, GISD and population density, and age and population density; for models of CFR, interactions of interest were between GISD and population density, and between incidence in the current wave and hospital beds per capita. Inclusion of interaction terms only improved model fit for the model of CFRs during wave 3. Because the model containing only the interaction between GISD and population density (and not the interaction between incidence and hospital beds) resulted in a lower BIC than the model with both interaction terms included, we retained only the interaction between GISD and population density in the final model.

Analysis of Partial Waves

In order to test whether there was evidence of substantial changes in the relationship between SEP and either incidence or CFR over the course of waves other than wave 1, we split each wave into two partial waves and refit the models described in the main text. Waves were divided as described above under "Defining Partial Pandemic Waves"

and in Table S1. All variables and interactions included in the full wave models were also included in the partial wave models. For the spatial smooths, we used the k-values chosen for each respective full wave, unless model checked using DHARMa or Moran's I indicated issues, in which case k-values for a given partial wave were updated accordingly. The associations between GISD and either incidence or CFR, controlling for all other variables, were calculated as described in the main text ("Model Assessment").

Age-Stratified Analysis

Although in the main text we used age standardization to consolidate cases and deaths from all age groups into a single measure of incidence and CFR for each district and wave, it is possible that key drivers of COVID-19 incidence and CFRs differ by age. In order to test this, we refit the models described in the main text to outcome measures calculated for individual age groups. To avoid fitting a large number of additional models, we chose a single age group for each outcome measure. Specifically, for models of incidence, we chose the 60+ age group. This is because many of our selected predictors, including the percentage of working-age adults and the number of care home beds per capita, may be expected to impact elderly adults differently than younger people. For CFRs, we chose those aged 15-59; the results in the main text likely reflect associations with CFR among the elderly, who made up the majority of COVID-19 deaths, but drivers of CFRs might be different in younger people.

As in the main analysis, cases and deaths with no associated age information were assigned to age groups proportionally to the age distribution of cases and deaths on the same day. For waves 3-5, vaccination rates for the specific age group of interest were included as predictors, rather than vaccination rates among the whole population. The k-values for all smooths were set to the values used in the main analysis, unless improved model fit was achieved with a different value.

Analysis Ignoring Spatial Dependence

Failure to adequately account for spatial dependence between regions can lead to biased results. In particular, confidence intervals may be too narrow, leading results to appear more certain than they really are. To demonstrate the effect of ignoring spatial dependence in our data, we fit all models described in the main text without the spatial smooth on latitude and longitude. All k-values for the remaining predictors were set to the values used in the main analysis.

COVID-19-Specific vs. Excess Mortality

Although we expect our mortality data to be high quality, if there are systematic regional differences in the extent of underreporting (driven, for example, by socioeconomic deprivation (38) or healthcare quality), the results of our CFR analyses could be biased. Analyzing excess deaths instead of COVID-19-specific deaths is one way to reduce this bias (39). Unfortunately, we do not have access to weekly, district-level all-cause mortality data. However, we were able to access weekly, state-level (n = 16) mortality data through Eurostat, the statistical office of the European Union (40). We used these data to assess the extent to which excess deaths and reported COVID-19 deaths displayed similar spatial patterns.

Excess deaths for each state and wave were calculated by subtracting the number of expected deaths during a given wave from the reported number of all-cause deaths during the same wave. Because the Eurostat data are reported by week of death, we shifted all wave definitions by two weeks, to account for the delay between infection with SARS-CoV-2 and death (41). In order to obtain the expected number of weekly deaths throughout the pandemic period, we fit a negative binomial GAM to the weekly mortality data from 2015 through 2019. Specifically, we included 1) a seasonal smooth on week of the year, with k = 53 and a cyclic cubic regression spline, such that there is no jump in the fit values between weeks 53 and 1 (35), to capture within-year trends in mortality, and 2) a linear effect of year, to capture secular trends in mortality. We fit a separate model for each state. This approach is similar to the one used in (42). As in (42), we assumed that expected deaths throughout the pandemic period were equal to the model-predicted weekly deaths for 2020; this was done to avoid over-extrapolating the long-term trends in mortality. We note that the Eurostat data are not age-specific, so we were unable to calculate age-standardized excess mortality.

Analysis of Pre-Pandemic Mortality

We obtained data on yearly, district-level all-cause mortality rates from INKAR (28). Because these data were reported as rates per 1000 population, we used yearly population counts and rounded to the nearest whole number to get the yearly number of deaths by district. Unfortunately, these data were not available by age, so no age-standardization was possible. We then fit a separate negative binomial GAM for each year from 2015 to 2019, with yearly deaths as the dependent variable and population size as the offset; as predictors, we included both the variables included in our models of incidence and those included in our models of CFRs (i.e., all variables listed in Table 1 with the exception of living space). As in the models described in the main text, appropriate values of k for the spatial smooths were chosen by assessing the resulting model residuals using the DHARMa package and Moran's I.

Supplementary Results

Analysis of Partial Waves

As in the full waves, both incidence and CFRs were significantly spatially clustered during each partial wave, with the exception of CFRs during wave 4.1; again, clustering was much stronger for incidence than for CFRs. There was a significant, positive correlation between the district-level cumulative incidence rates during the two partial waves for waves 1 through 3, but not for waves 4 and 5, indicating that differences in the spatial patterns in incidence occurred during these last two waves. Significant correlations between partial waves for CFR were observed for all waves except wave 4.

Fitted associations between GISD scores and district-level COVID-19 incidence and CFRs for partial waves 2.1 through 5.2 can be seen in Figure S7. For all waves, the relationship between GISD and incidence was similar for both partial waves (Figure S7a). Thus, we found no evidence for a change in the direction of the association between GISD and incidence partway through wave 2, as was reported in (25). Similar results were found for CFRs, with the exception of wave 4, where there was evidence of a negative association with socioeconomic deprivation in partial wave 1 and a positive association in partial wave 2. However, as the majority of COVID-19 cases during wave 4 occurred during partial wave 4.2 (Figure S1), we retained the original analysis of the full wave in the main text.

Age-Stratified Analysis

As in the overall population, COVID-19 incidence was by far the highest during wave 5, and lowest during wave 1 (Table S2). Relative to the full, age-standardized population, individuals aged 60 and above reported significantly lower rates of infection in all waves except wave 1.2, where incidence in this age group was slightly but significantly higher. Differences in incidence became more substantial as the pandemic progressed. This is consistent with reports that later pandemic waves were dominated by younger age groups. Spatial clustering in incidence, however, was similar to clustering among the whole population across all waves (Table S2).

Compared to the age-standardized population, individuals aged 15 through 59 experienced significantly lower CFRs across all waves, with a particularly large difference early in the pandemic (Table S3). This was expected, given that death following infections with SARS-CoV-2 were predominantly reported in elderly individuals (43). Spatial clustering in CFRs was similar to that seen in the full, age-standardized population in waves 1 and 2, but significantly lower in waves 3-5 (Table S3). Unlike in the full population, district-level cumulative CFRs were not correlated across waves, indicating a lack of a conserved pattern in CFRs over time.

Table S2. Observed cumulative incidence (per 10,000 population) among individuals aged 60+ for the first five waves of COVID-19 in Germany, as well as the extent of observed spatial clustering, as measured by Moran's I. A "*" next to the reported incidence range indicates a significant difference from the incidence range reported by the full, age-standardized population, as measured using a Wilcoxon signed-rank test, with a Bonferroni-adjusted p-value cutoff of 0.05/12 = 0.004.

Pandemic Wave		Median Incidence (Range)	Moran's I (95% CI)
1	1,1	7.80 (0.80, 134.9)*	0.48 (0.42, 0.55)
	1.2	8.30 (0.14, 104.6)*	0.48 (0.42, 0.55)
2		215.0 (46.0, 667.2)*	0.68 (0.61, 0.74)
3		76.7 (14.8, 307.5)*	0.67 (0.61, 0.74)
4		174.6 (40.9, 660.3)*	0.86 (0.79, 0.92)
5		1067.2 (647.6, 1904.7)*	0.66 (0.60, 0.73)

Table S3. Observed cumulative case fatality rates (CFRs; %) among individuals aged 15 through 59 for the first five waves of COVID-19 in Germany, as well as the extent of observed spatial clustering, as measured by Moran's I. A "*" next to the reported CFR range indicates a significant difference from the CFR range reported by the full, age-standardized population, as measured using a Wilcoxon signed-rank test, with a Bonferroni-adjusted p-value cutoff of 0.05/12 = 0.004.

Pandemic Wave		Median CFR (Range)	Moran's I (95% CI)
1	1,1	0 (0, 10.0)*	-0.030 (-0.090, 0.030)
	1.2	0 (0, 14.29)*	-0.007 (-0.068, 0.054)
2		0.15 (0, 0.95)*	0.080 (0.015, 0.14)
3		0.10 (0, 0.82)*	0.10 (0.039, 0.17)
4		0.15 (0, 0.96)*	0.21 (0.15, 0.28)
5		0.08 (0, 0.65)*	0.14 (0.070, 0.20)

Results of the age-stratified analyses can be found in Figures S9 (for the models of incidence) and S10 (for the models of CFR). For some waves and predictors, there are indeed differences in the associations identified. For example, there appears to be a slight negative association between the percentage of workers in service jobs and incidence among the 60+ age group in wave 1.2, which is not present for the full population (Figure S8). Perhaps most notably, while we found a negative association between socioeconomic deprivation and CFRs in the full population during wave 1.2, we instead see a positive association when looking only at those aged 15-59. However, for the vast majority of waves and predictors, there is very little difference between the age-specific and full population results. Whether the drivers of incidence and CFRs are actually similar across age groups, or whether the data are simply not of sufficient quality to allow for age-specific drivers to be identified, is unclear.

Analysis Ignoring Spatial Dependence

Figure S10 shows the inferred association between socioeconomic deprivation and both incidence and CFRs due to COVID-19 found when ignoring latitude and longitude, in comparison to the same results as found in the main analysis. Although the strength and direction of the effect of deprivation is similar between the two analyses, the confidence intervals are narrower when no spatial smooth is included, as expected for models fit to data with spatial autocorrelation. This is particularly true for the models fit to incidence data, which are more spatially structured than the data on CFRs. In contrast, no difference in the confidence intervals is observed for the models of CFR during waves 1 and 5, as there was no remaining effect of the smooth over latitude and longitude on CFR during these waves after controlling for all other variables. Furthermore, there is significant spatial autocorrelation among the residuals for all models except those of CFRs in waves 1 and 4, indicating that the assumption of independence among the residuals is violated for these models.

COVID-19-Specific vs. Excess Mortality

We found that reported COVID-19 deaths and excess deaths were highly correlated at the state level (Pearson's $\rho = 0.76$, $p < 0.001$; Figure S11), suggesting that the death data used in the main text accurately capture the spatial patterns in relative COVID-19-induced mortality, at least at this spatial scale. We note that, although we have plotted the data on a log scale to more clearly visualize the association, the test of correlation was conducted using the raw (i.e., not transformed) data. Furthermore, we found that the number of COVID-specific deaths is almost always greater than the number of excess deaths in all waves except wave 4, suggesting that COVID-19 deaths were not generally underreported in Germany. Interestingly, wave 4 is also the only wave in which excess deaths were greater than 0 in all states, perhaps reflecting the impact of various COVID-19 control measures on mortality due to other causes.

Analysis of Pre-Pandemic Mortality

We find that increasing deprivation is associated with increased all-cause mortality in all five years considered, and that the magnitude of this association is very similar year to year (Figure S12). This is in clear contrast to our findings regarding incidence and CFRs due to COVID-19 (main text Figures 1 and 2), where both the magnitude and direction of the association with deprivation vary considerably over time. The strength of the pre-pandemic associations also tended to be weaker than the positive associations between deprivation and COVID-19-specific outcomes, in particular for incidence in waves 1 and 3, and for CFRs in wave 5, although we note that credible intervals for the main text results are quite wide. As in the main text, we emphasize that, because the outcome measures in our main text analyses are not measures that can be calculated for the pre-pandemic period, these results are not directly comparable, and conclusions should be drawn with caution. As a final caveat, we note again that all-cause mortality data were not available by age; although we attempt to control for the proportion of each district belonging to different age groups, it is likely that some effect of varying age structure remains.

Supplementary Figures

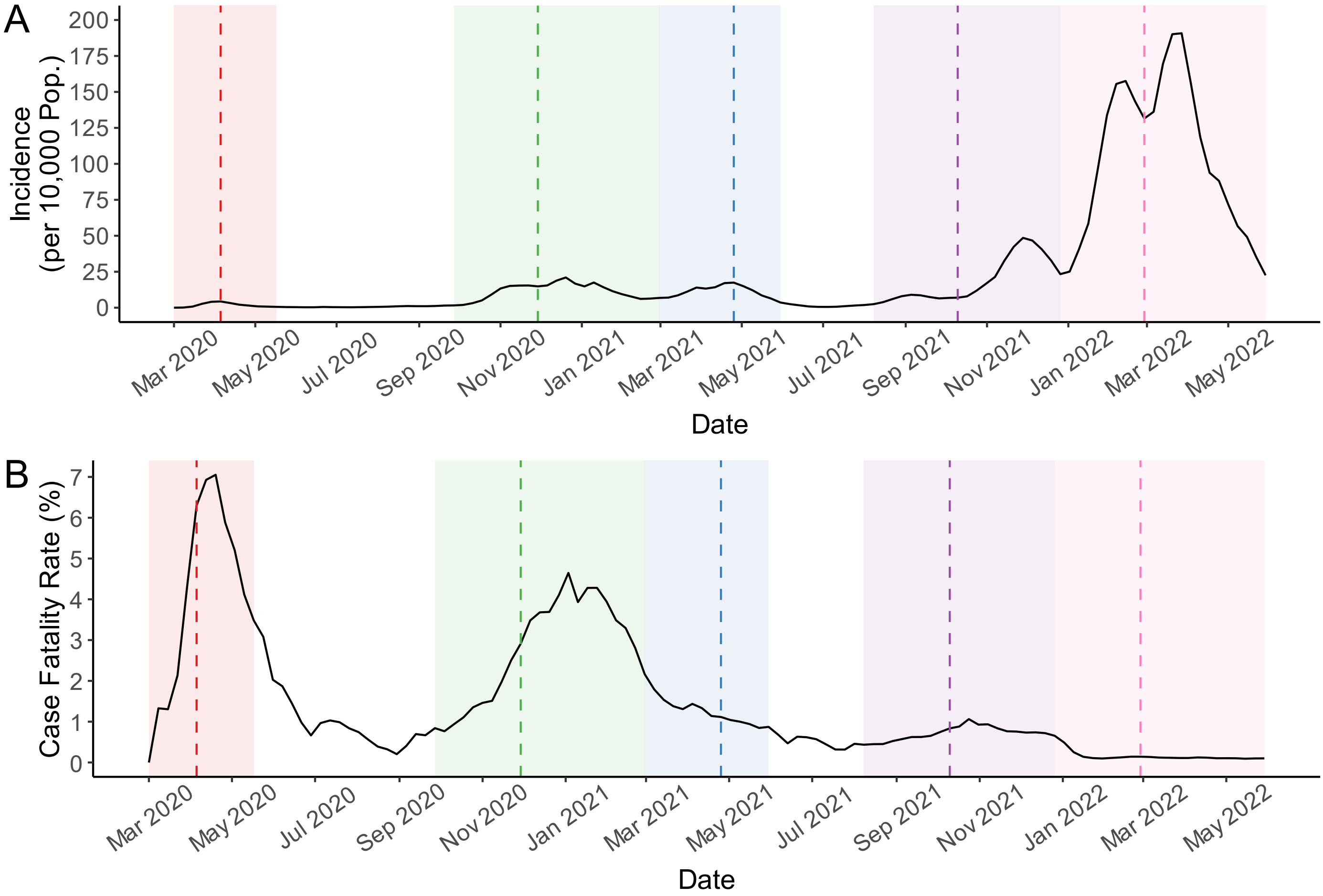

Figure S1. Country-level COVID-19 incidence (A) and CFRs (B) during the first five pandemic waves in Germany. Shaded boxes denote the five pandemic waves, with colors chosen as in main text Figure 1 (red = wave 1, green = wave 2, blue = wave 4, purple = wave 4, pink = wave 5). Dotted vertical lines show where waves were divided into partial waves.

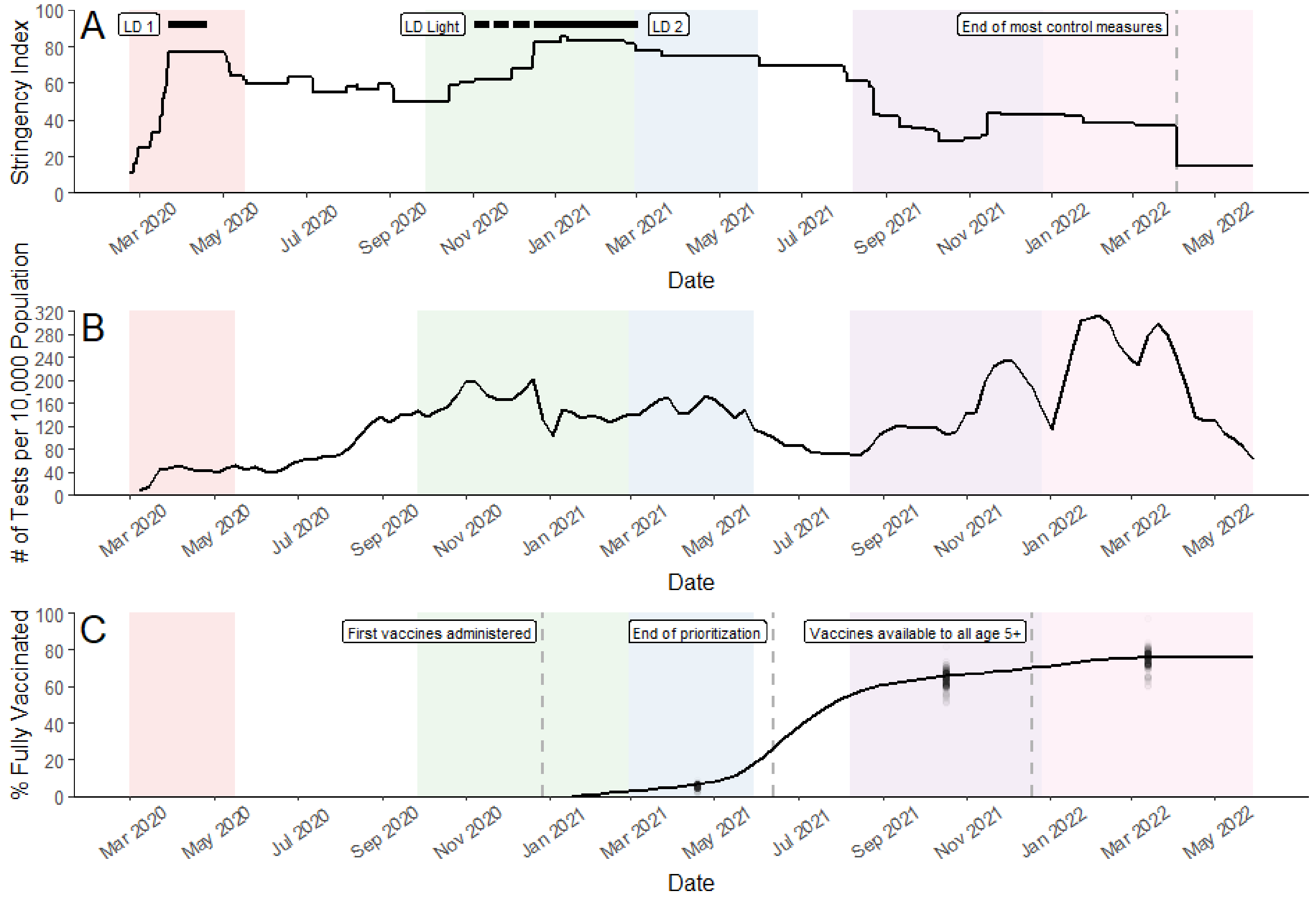

Figure S2. Timeline of the government response to the COVID-19 pandemic in Germany, including intensity of country-level control measures (A), testing effort (B), and vaccine policy (C). Colors represent the time periods for each wave, as in Figure S1. Significant timepoints are denoted by vertical gray dashed lines and labeled accordingly; the time periods of the three major national lockdowns ("LD") are shown in (A) as horizontal black lines. In (C), the range of district-level vaccination coverage rates at the midpoint of waves 3, 4, and 5 are also shown.

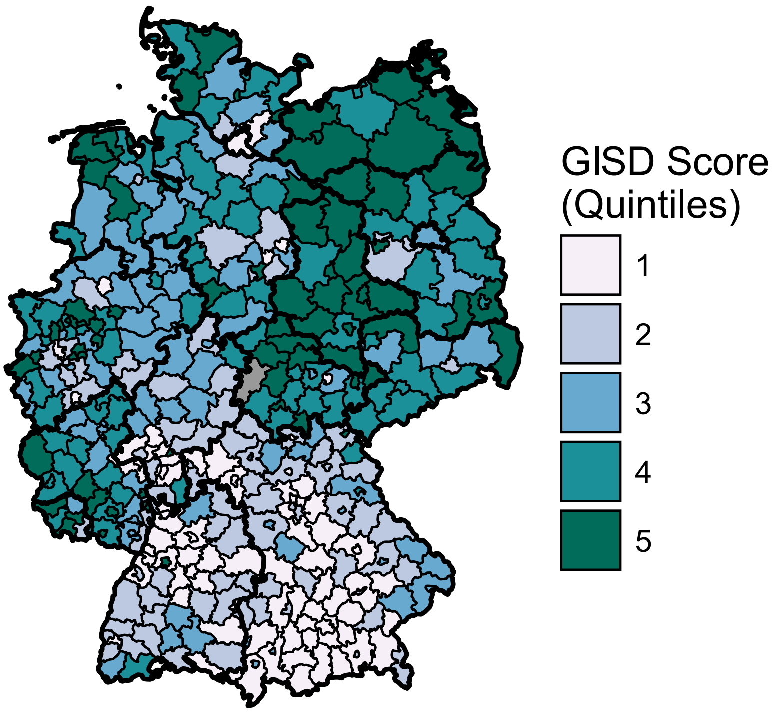

Figure S3. GISD scores (as quintiles) for each district in Germany. The thicker black lines represent the borders of the 16 federal states. Note that higher values of the GISD indicate higher deprivation, i.e., lower socioeconomic position. The merged district that was removed from the data prior to analysis is shown in dark gray. Map data for district and state boundaries in Germany were obtained from the Federal Agency for Cartography and Geodesy (44), © GeoBasis-DE / BKG (2022).

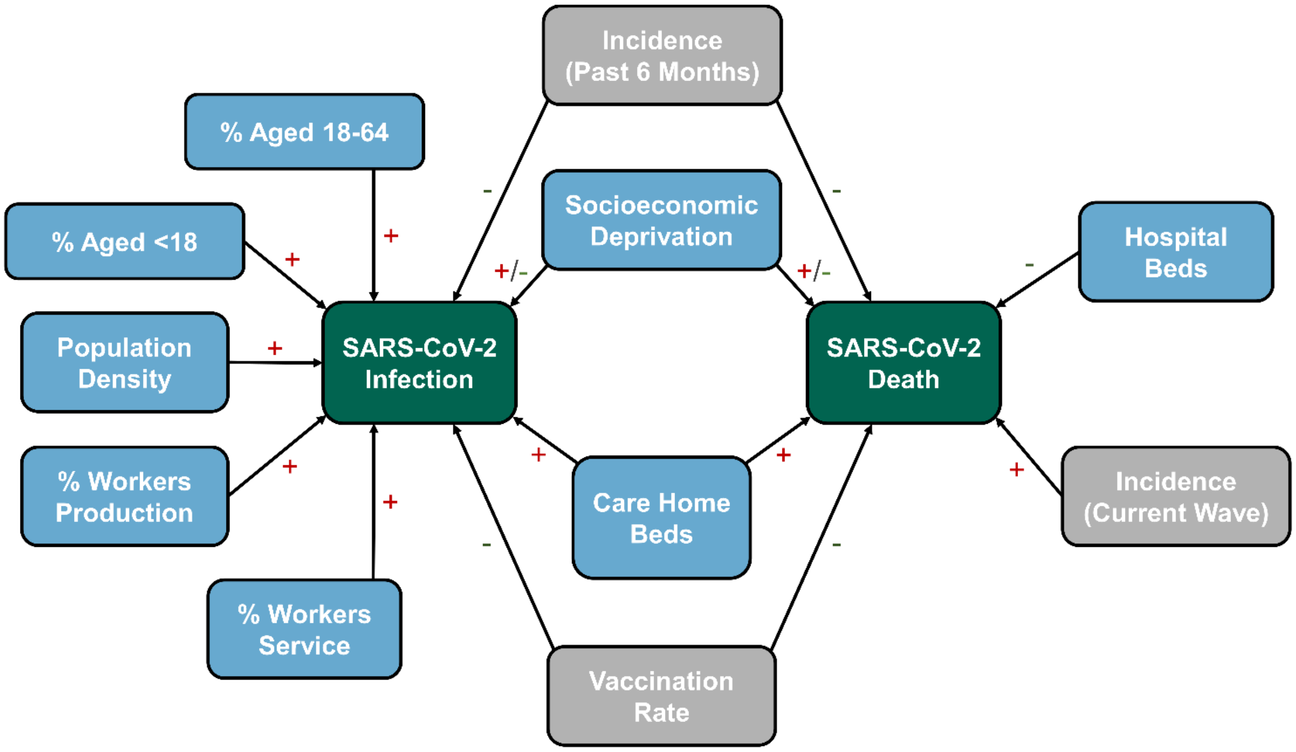

Figure S4. Directed acyclic graph depicting the hypothesized causal links between COVID-19 cases and deaths and various socioeconomic, demographic, and healthcare-related predictors. Dependent variables are shown in dark green boxes, key predictors are represented by blue boxes, and additional predictors are shown in gray. Arrows from predictors hypothesized to increase incidence or CFRs are labeled with a red “+,” whereas arrows from predictors hypothesized to decreases incidence or CFRs are labeled with a green “-.” Because both positive and negative relationships between SEP and incidence have been observed over time in Germany, the arrow from GISD is labeled with both a “+” and a “-.”

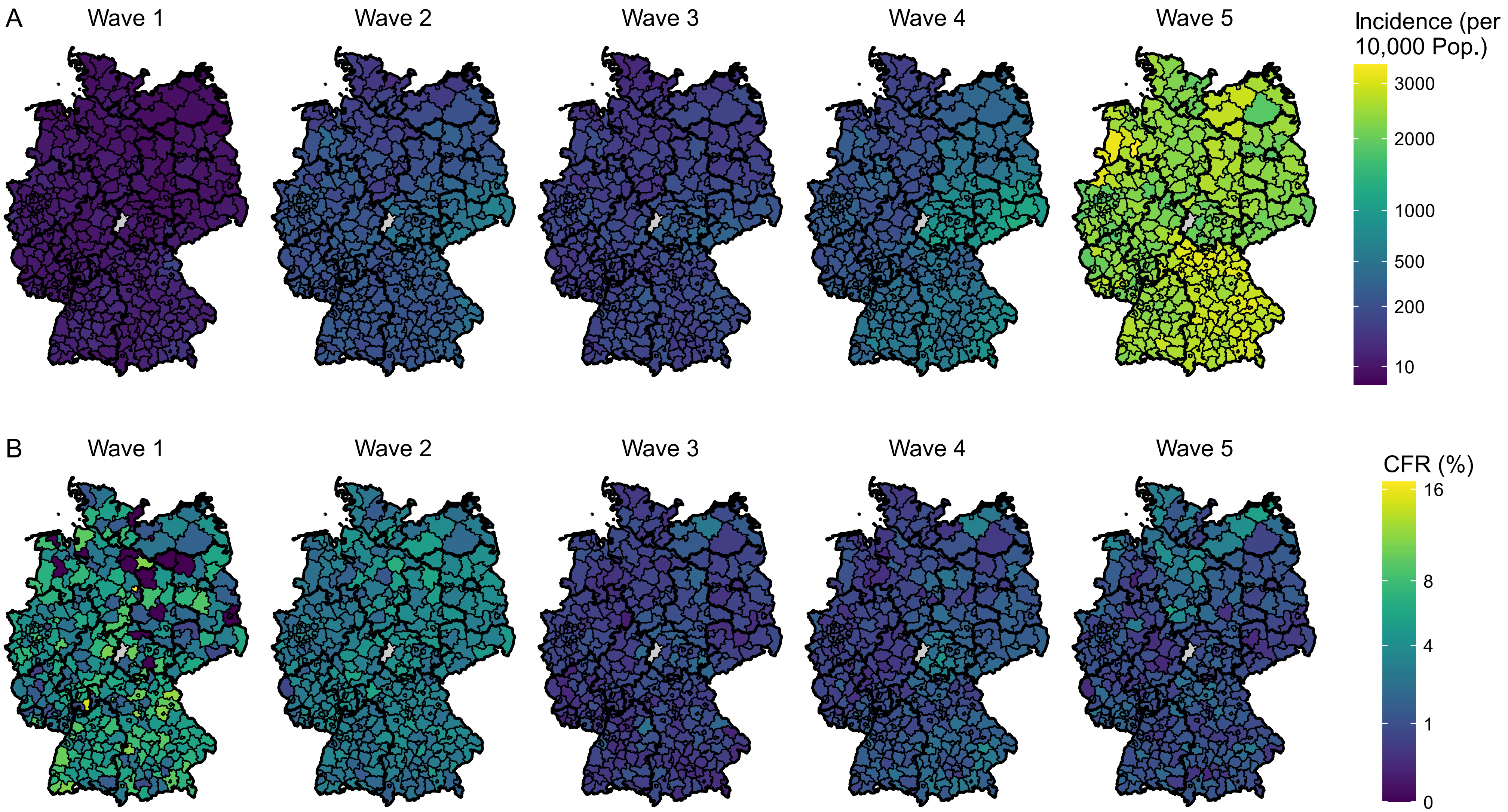

Figure S5. Observed cumulative district-level COVID-19 incidence per 10,000 population (A) and case fatality rates (B) in Germany during the first five waves of the pandemic. The merged district that was removed from the data prior to analysis is shown in gray. Map data for district and state boundaries in Germany were obtained from the Federal Agency for Cartography and Geodesy (44), © GeoBasis-DE / BKG (2022).

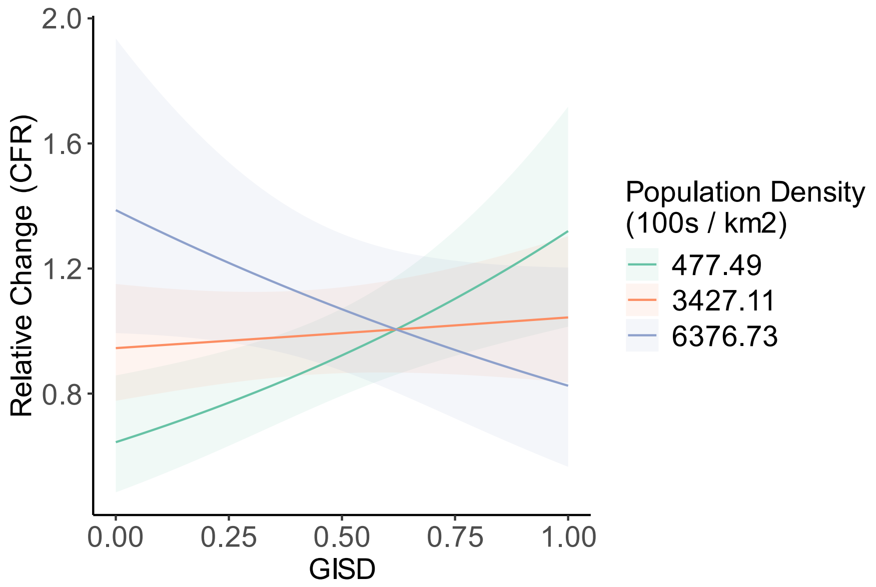

Figure S6. Predicted multiplicative change in COVID-19 CFRs by GISD score and population density, for wave 3. As in main text Figure 2, solid lines represent the marginal effect of GISD relative to the mean effect size, and shaded areas represent 95% confidence intervals. Predictions are shown separately for three representative levels of population density: the minimum population density observed in the data (in green), the maximum observed population density (in purple), and the midpoint between the minimum and maximum observed values (in orange).

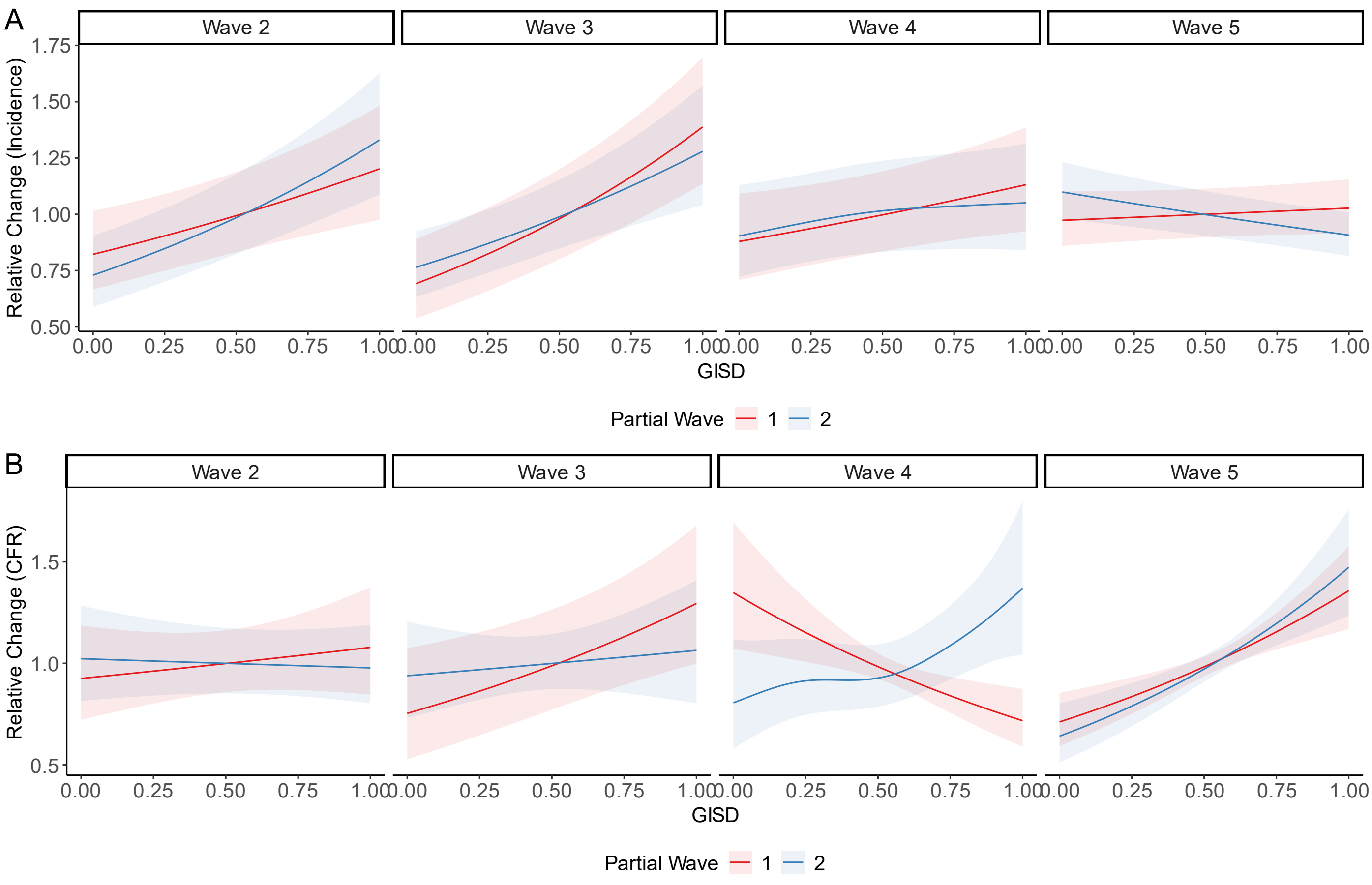

Figure S7. Predicted multiplicative change in COVID-19 incidence (A) and CFRs (B) with changing GISD score, by partial wave. Solid lines represent the marginal effect of GISD; shaded areas represent 95% confidence intervals. For each wave, results from the first partial wave are shown in red, and from the second partial wave are shown in blue.

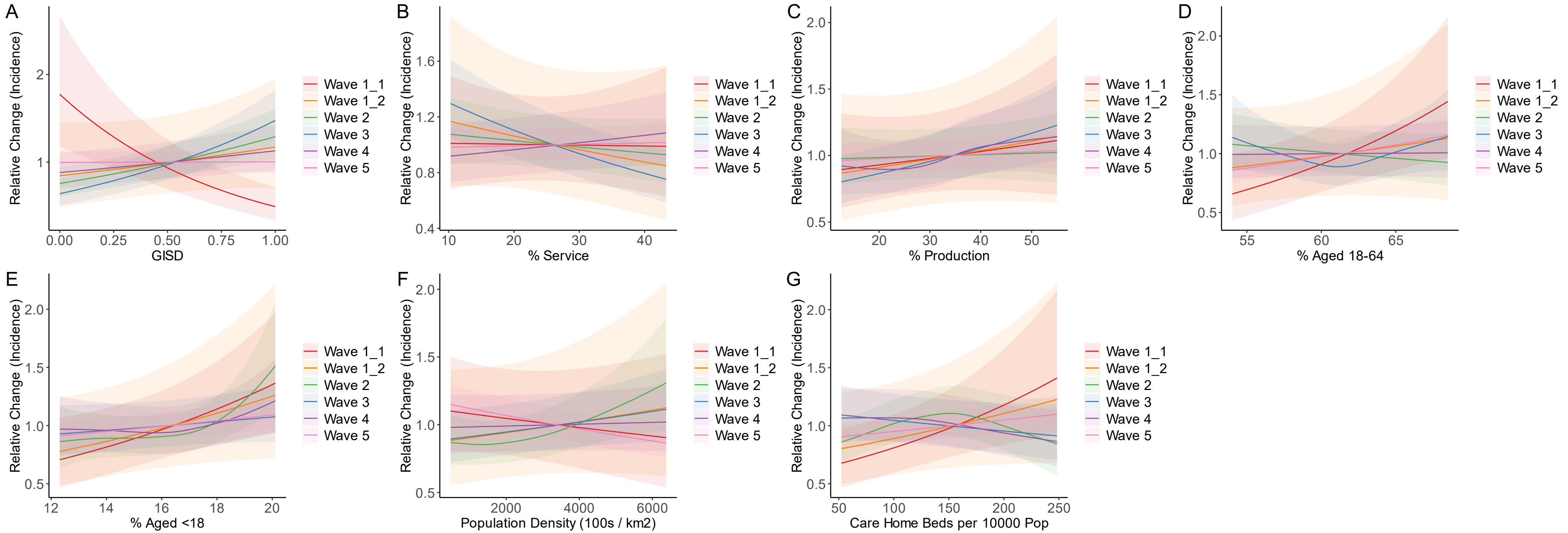

Figure S8. Predicted multiplicative change in COVID-19 incidence among those aged 60 and above with changing values of district-level predictors, by pandemic wave. Solid lines represent the marginal effect of each predictor when all other predictors are set to their mean values, and shaded areas represent 95% confidence intervals. The predictor variables shown in (A) through (G) are as in Figure 1.

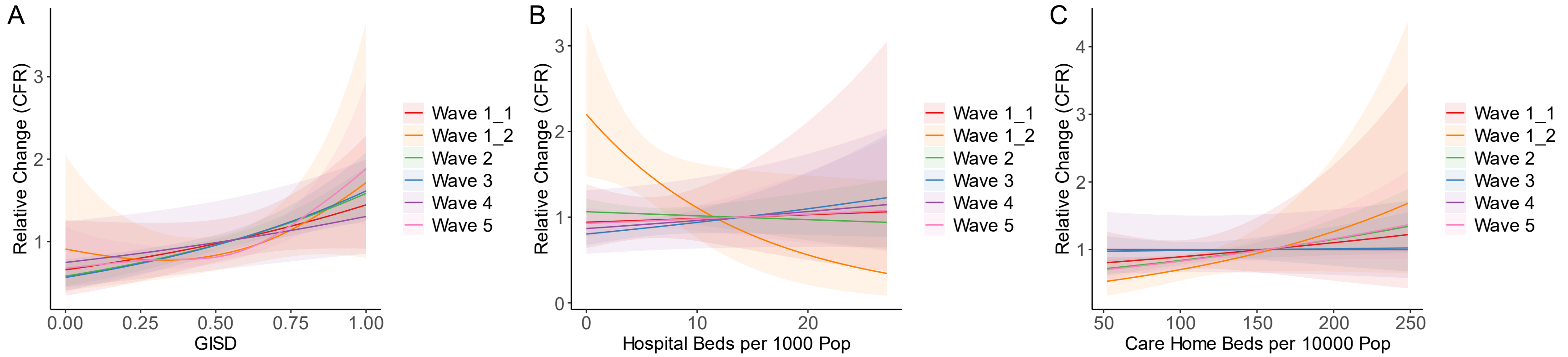

Figure S9. Predicted multiplicative change in COVID-19 CFRs among those aged 15-59 with changing values of district-level predictors, by pandemic wave. Solid lines represent the marginal effect of each predictor when all other predictors are set to their mean values, and shaded areas represent 95% confidence intervals. The predictor variables shown in (A) through (C) are as in Figure 2.

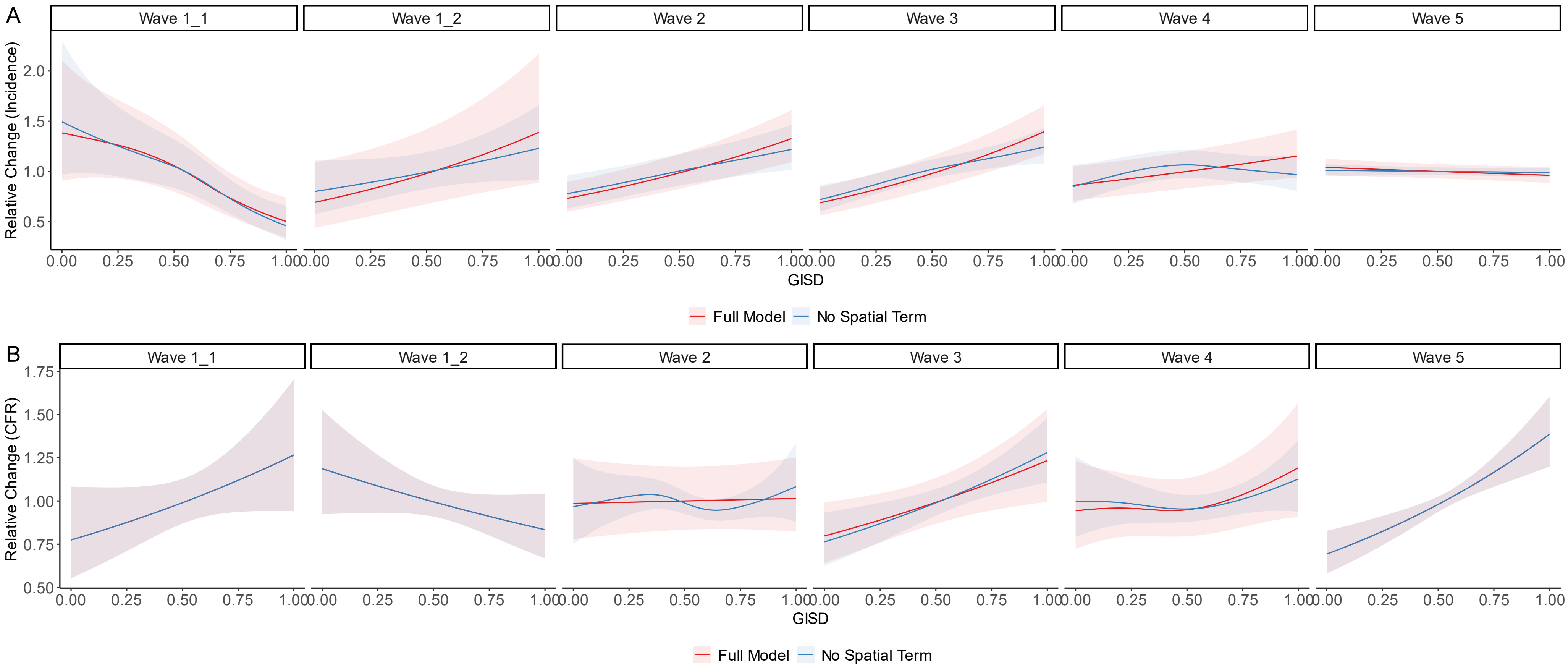

Figure S10. The effect of excluding the spatial smooth on the inferred association between GISD and COVID-19 incidence (A) and CFRs (B), by wave. Solid lines represent the predicted, multiplicative effect of GISD when all other predictors are set to their mean values; shaded areas represent 95% confidence intervals. Results for models including a two-dimensional smooth of latitude and longitude (as described in the main text) are shown in red, while results from models with no spatial smooth are shown in blue.

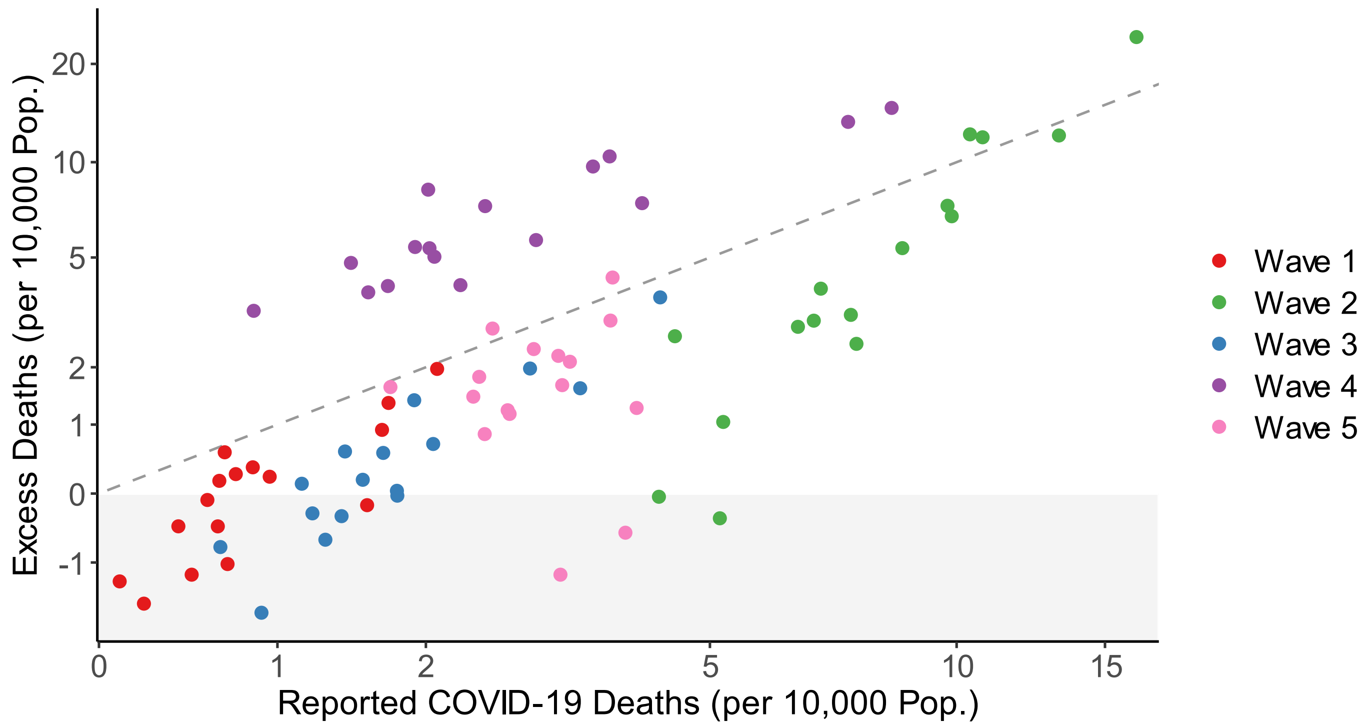

Figure S11. Association between state-level reported COVID-19 deaths and excess deaths. The gray dotted line represents equivalence between reported COVID-19 and calculated excess deaths, while the shaded gray area shows where excess deaths are negative. Points are colored according to pandemic wave.

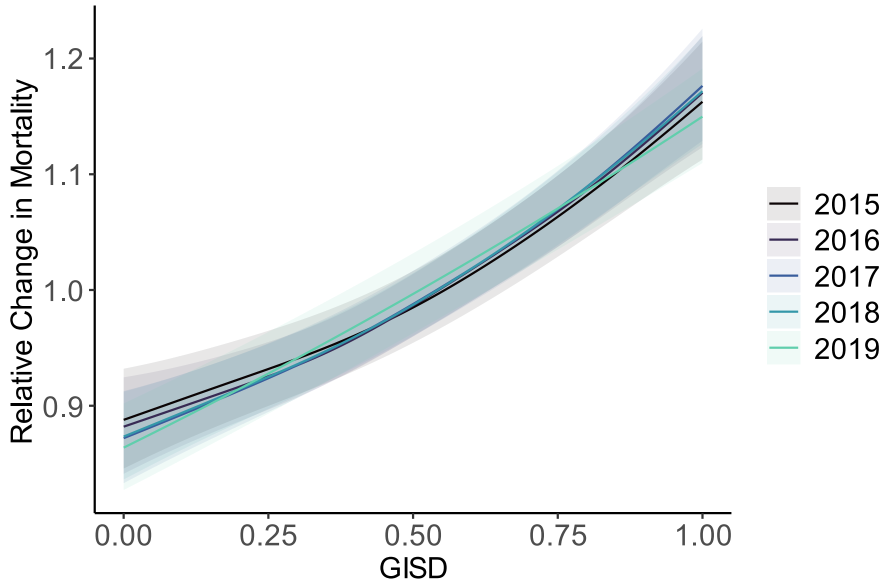

Figure S12. Predicted multiplicative change in pre-pandemic all-cause mortality rates with changing GISD score, by year. Solid lines represent the marginal effect of GISD when all other predictors are set to their mean values, and the shaded areas represent 95% confidence intervals.

References

1. Hale T, Angrist N, Goldszmidt R, Kira B, Petherick A, Phillips T, et al. A global panel database of pandemic policies (Oxford COVID-19 Government Response Tracker). Nat Hum Behav. 2021 Apr;5(4):529–38.

2. Thurau J, Bosen R. Chronologie: Corona in Deutschland. Deutsche Welle [Internet]. 2021 Jun 23 [cited 2025 Apr 7]; Available from: https://www.dw.com/de/chronologie-ausbreitung-des-coronavirus-in-deutschland/a-58003172

3. Telefonkonferenz der Bundeskanzlerin mit den Regierungschefinnen und Regierungschefs der Länder am 13. Dezember 2020 [Internet]. 2020 Dec. Available from: https://www.bundesregierung.de/resource/blob/997532/1827366/69441fb68435a7199b3d3a89bff2c0e6/2020-12-13-beschluss-mpk-data.pdf?download=1

4. WELT. Corona: Merkel sieht „Grund zur Beunruhigung“ – Das ist die neue „Hotspot-Strategie“. Die Welt [Internet]. 2020 Sep 29 [cited 2025 Apr 7]; Available from: https://www.welt.de/politik/deutschland/article216846804/Corona-Merkel-sieht-Grund-zur-Beunruhigung-Das-ist-die-neue-Hotspot-Strategie.html

5. Mit klaren Regelungen bei steigenden Infektionszahlen verstärkt Nordrhein-Westfalen die Schutzmaßnahmen in der Corona-Pandemie [Internet]. [cited 2025 Apr 7]. Available from: https://www.land.nrw/pressemitteilung/mit-klaren-regelungen-bei-steigenden-infektionszahlen-verstaerkt-nordrhein

6. Die Bundesregierung informiert | Startseite [Internet]. [cited 2025 Apr 3]. Homeoffice-Regelung verlängert. Available from: https://www.bundesregierung.de/breg-de/service/archiv/verordnung-zu-homeoffice-1841120

7. Arbeit - 3G am Arbeitsplatz und Homeoffice: Was ab Mittwoch gilt. Süddeutsche Zeitung [Internet]. 2021 Nov 23 [cited 2025 Apr 7]; Available from: https://www.sueddeutsche.de/karriere/arbeit-3g-am-arbeitsplatz-und-homeoffice-was-ab-mittwoch-gilt-dpa.urn-newsml-dpa-com-20090101-211123-99-109188

8. Thurau J. Die neuen 3G-Regeln in Deutschland. Deutsche Welle [Internet]. 2021 Aug 23 [cited 2025 Apr 3]; Available from: https://www.dw.com/de/genesen-geimpft-oder-getestet-die-neuen-3g-regeln-in-deutschland/a-58957331

9. Knecht J. 3G-Regel, 2G-Regel, 2G Plus-Regel: Unterschied und Bedeutung der Corona-Regeln. FOCUS online [Internet]. 2021; Available from: https://praxistipps.focus.de/3g-regel-2g-regel-2g-plus-regel-unterschied-und-bedeutung-der-corona-regeln_136926

10. Schmoll H. Corona: Die Maskenpflicht und was der Verstoß dagegen kostet. Frankfurter Allgemeine Zeitung [Internet]. 2020 Apr 27 [cited 2025 Apr 7]; Available from: https://www.faz.net/aktuell/politik/inland/corona-die-maskenpflicht-und-was-der-verstoss-dagegen-kostet-16742770.html

11. Connor R, Silk J. Germany extends COVID lockdown until February 14. Deutsche Welle [Internet]. 2021 Jan 19 [cited 2025 Apr 7]; Available from: https://www.dw.com/en/coronavirus-germany-extends-covid-lockdown-until-february-14/a-56277168

12. Deutsche Welle. Germany lifts most COVID-19 restrictions. Deutsche Welle [Internet]. 2022 Mar 18 [cited 2025 Apr 3]; Available from: https://www.dw.com/en/germany-lifts-most-covid-19-restrictions-after-difficult-compromise/a-61174654

13. Deutsche Welle. Germany: Some COVID rules eased, but “Freedom Day” delayed. Deutsche Welle [Internet]. 2022 Mar 20 [cited 2025 Apr 3]; Available from: https://www.dw.com/en/germany-some-covid-rules-eased-but-states-delay-freedom-day/a-61189258

14. Haserück A. SARS-CoV-2-Diagnostik: Testempfehlungen sind angepasst worden. Dtsch Arztebl [Internet]. 2020;46. Available from: https://www.aerzteblatt.de/archiv/sars-cov-2-diagnostik-testempfehlungen-sind-angepasst-worden-b65a298d-9e55-44da-9f94-597499521f21

15. Richter-Kuhlmann E, Maybaum T. Neue Testverordnung: PCR-Test nur noch nach positivem Antigenschnelltest. Dtsch Arztebl [Internet]. 2022;7. Available from: https://www.aerzteblatt.de/archiv/neue-testverordnung-pcr-test-nur-noch-nach-positivem-antigenschnelltest-8352ae28-cef1-4d3d-b25f-bd33eaaa5a28

16. Verordnung zum Anspruch auf Schutzimpfung gegen das Coronavirus SARS-CoV-2 [Internet]. Feb 8, 2021. Available from: https://www.bundesgesundheitsministerium.de/service/gesetze-und-verordnungen/detail/coronavirus-impfverordnung-coronaimpfv.html

17. Gera V, Mchugh D. Germany, Hungary give 1st vaccine shots ahead of EU rollout. AP News [Internet]. 2020 Dec 26 [cited 2025 Apr 7]; Available from: https://apnews.com/article/europe-italy-poland-coronavirus-pandemic-coronavirus-vaccine-813694862082592098e9915cdf85cb80

18. Corona-Impfung für alle ab 7. Juni möglich. Der Spiegel [Internet]. 2021 May 17 [cited 2025 Apr 3]; Available from: https://www.spiegel.de/politik/deutschland/jens-spahn-corona-impfung-fuer-alle-ab-7-juni-moeglich-a-dacb2759-34f5-49ea-802c-4a292ff10ff0

19. Deutsche Welle. EU regulators approve COVID vaccine for children. Deutsche Welle [Internet]. 2021 May 28 [cited 2025 Apr 3]; Available from: https://www.dw.com/en/eu-regulators-approve-biontech-pfizer-covid-vaccine-for-children/a-57697157

20. Berlin, other German states offer COVID-19 vaccine to 5-11s. Independent [Internet]. 2021 Dec 12 [cited 2025 Apr 3]; Available from: https://www.independent.co.uk/news/children-berlin-covid-vienna-pfizer-b1974553.html

21. Schilling J, Tolksdorf K, Marquis A, Faber M, Pfoch T, Buda S, et al. Die verschiedenen Phasen der COVID-19-Pandemie in Deutschland: Eine deskriptive Analyse von Januar 2020 bis Februar 2021. Bundesgesundheitsblatt - Gesundheitsforschung - Gesundheitsschutz. 2021 Sep 1;64(9):1093–106.

22. Plümper T, Neumayer E. The pandemic predominantly hits poor neighbourhoods? SARS-CoV-2 infections and COVID-19 fatalities in German districts. Eur J Public Health. 2020 Dec 11;30(6):1176–80.

23. Hoebel J, Michalski N, Diercke M, Hamouda O, Wahrendorf M, Dragano N, et al. Emerging socio-economic disparities in COVID-19-related deaths during the second pandemic wave in Germany. Int J Infect Dis. 2021 Dec;113:344–6.

24. Robert Koch Institute. Wöchentlicher Lagebericht des RKI zur Coronavirus-Krankheit-2019 (COVID-19) [Internet]. Robert Koch Institute; 2022. Available from: https://www.rki.de/DE/Content/InfAZ/N/Neuartiges_Coronavirus/Situationsberichte/Wochenbericht/Wochenbericht_2022-01-27.pdf?__blob=publicationFile

25. Hoebel J, Michalski N, Wachtler B, Diercke M, Neuhauser H, Wieler LH, et al. Socioeconomic Differences in the Risk of Infection During the Second Sars-Cov-2 Wave in Germany. Dtsch Arztebl Int. 2021 Apr 16;118(15):269–70.

26. Ahmad OB, Boschi-Pinto C, Lopez AD, Murray C, Lozano R, Inoue M. Age standardization of rates: A new who standard. 2000; Available from: https://www.academia.edu/download/31226140/3_WHO_pop_standard.pdf

27. Michalski N, Reis M, Tetzlaff F, Herber M, Kroll LE, Hövener C, et al. German Index of Socioeconomic Deprivation (GISD): Revision, update and applications. J Health Monit. 2022 Dec;7(Suppl 5):2–23.

28. Bundesinstituts für Bau-, Stadt- und Raumforschung (BBSR). INKAR - Indikatoren und Karten zur Raum- und Stadtentwicklung [Internet]. 2024. Available from: https://www.inkar.de

29. Kroll L, Schumann M, Hoebel J, Lampert T. Regionale Unterschiede in der Gesundheit – Entwicklung eines sozioökonomischen Deprivationsindex für Deutschland. 2017 Jun 14; Available from: https://edoc.rki.de/handle/176904/2648

30. Thompson MG, Stenehjem E, Grannis S, Ball SW, Naleway AL, Ong TC, et al. Effectiveness of Covid-19 Vaccines in Ambulatory and Inpatient Care Settings. N Engl J Med. 2021 Oct 7;385(15):1355–71.

31. Tenforde MW, Olson SM, Self WH, Talbot HK, Lindsell CJ, Steingrub JS, et al. Effectiveness of Pfizer-BioNTech and Moderna Vaccines Against COVID-19 Among Hospitalized Adults Aged ≥65 Years - United States, January-March 2021. MMWR Morb Mortal Wkly Rep. 2021 May 7;70(18):674–9.

32. Robert Koch-Institut, Fachgebiet. COVID-19-Impfungen in Deutschland [Internet]. Zenodo; 2022. Available from: https://zenodo.org/record/7084866

33. Pebesma E, Bivand R. Spatial Data Science: With Applications in R. Boca Raton: Chapman and Hall/CRC; 2023.

34. Pebesma E. Simple features for R: Standardized support for spatial vector data. R J. 2018;10(1):439.

35. Wood SN. Generalized Additive Models: An Introduction with R, Second Edition. CRC Press; 2017. 496 p.

36. Hartig F. DHARMa: Residual Diagnostics for Hierarchical (Multi-Level / Mixed) Regression Models [Internet]. 2022. Available from: https://CRAN.R-project.org/package=DHARMa

37. Wood SN. Thin plate regression splines. J R Stat Soc Series B Stat Methodol [Internet]. 2003; Available from: https://academic.oup.com/jrsssb/article-abstract/65/1/95/7110632

38. Stokes AC, Lundberg DJ, Elo IT, Hempstead K, Bor J, Preston SH. COVID-19 and excess mortality in the United States: A county-level analysis. PLoS Med. 2021 May;18(5):e1003571.

39. Vandenbroucke JP, Pearce N. Excess mortality calculations to assess the impact of the COVID-19 pandemic: Concepts and methodological issues. Am J Public Health. 2024 Jun;114(6):593–8.

40. Eurostat. Weekly death statistics [Internet]. [cited 2025 Mar 18]. Available from: https://ec.europa.eu/eurostat/statistics-explained/index.php?title=Weekly_death_statistics

41. von Stillfried S, Bülow RD, Röhrig R, Boor P, German Registry of COVID-19 Autopsies (DeRegCOVID), DeRegCOVID Collaborators. First report from the German COVID-19 autopsy registry. Lancet Reg Health Eur. 2022 Apr;15:100330.

42. Karlinsky A, Kobak D. Tracking excess mortality across countries during the COVID-19 pandemic with the World Mortality Dataset. Elife [Internet]. 2021 Jun 30;10. Available from: http://dx.doi.org/10.7554/eLife.69336

43. O'Driscoll M, Ribeiro Dos Santos G, Wang L, Cummings DAT, Azman AS, Paireau J, et al. Age-specific mortality and immunity patterns of SARS-CoV-2. Nature. 2021 Feb;590(7844):140–5.

44. Bundesamt für Kartographie und Geodäsie. Verwaltungsgebiete 1:2 500 000, Stand 31.12. (VG2500 31.12.) [Internet]. 2022. Available from: https://gdz.bkg.bund.de/index.php/default/open-data/verwaltungsgebiete-1-2-500-000-stand-31-12-vg2500-12-31.html
